# Population‐level effectiveness of pre‐exposure prophylaxis for HIV prevention among men who have sex with men in Montréal (Canada): a modelling study of surveillance and survey data

**DOI:** 10.1002/jia2.26194

**Published:** 2023-12-06

**Authors:** Carla M. Doyle, Rachael M. Milwid, Joseph Cox, Yiqing Xia, Gilles Lambert, Cécile Tremblay, Joanne Otis, Marie‐Claude Boily, Jean‐Guy Baril, Réjean Thomas, Alexandre Dumont Blais, Benoit Trottier, Daniel Grace, David M. Moore, Sharmistha Mishra, Mathieu Maheu‐Giroux

**Affiliations:** ^1^ Department of Epidemiology and Biostatistics School of Population and Global Health McGill University Montréal Québec Canada; ^2^ Direction Régionale de Santé Publique de Montréal Montréal Québec Canada; ^3^ Clinical Outcomes Research and Evaluation Research Institute ‐ McGill University Health Centre Montréal Québec Canada; ^4^ Centre de Recherche du Centre Hospitalier de l'Université de Montréal (CRCHUM) Montréal Québec Canada; ^5^ Département de Microbiologie Infectiologie et Immunologie Université de Montréal Montréal Québec Canada; ^6^ Département de Sexologie Université du Québec à Montréal Montréal Québec Canada; ^7^ MRC Centre for Global Infectious Disease Analysis School of Public Health Imperial College London London UK; ^8^ Department of Family Medicine Centre Hospitalier de l'Université de Montréal Montréal Québec Canada; ^9^ Clinique de médecine urbaine du Quartier Latin Montréal Québec Canada; ^10^ Clinique médicale l'Actuel Montréal Québec Canada; ^11^ RÉZO Health and STI prevention for GBQ men, trans people and MSM Montréal Québec Canada; ^12^ Dalla Lana School of Public Health University of Toronto Toronto Ontario Canada; ^13^ BC Centre for Excellence in HIV/AIDS Vancouver British Columbia Canada; ^14^ Faculty of Medicine University of British Columbia Vancouver British Columbia Canada; ^15^ Department of Medicine St. Michael's Hospital University of Toronto Toronto Ontario Canada; ^16^ Institute of Medical Sciences University of Toronto Toronto Ontario Canada; ^17^ Institute of Health Policy Management and Evaluation Dalla Lana School of Public Health University of Toronto Toronto Ontario Canada

**Keywords:** antiretroviral, combination prevention, elimination, epidemiology, impact evaluation, mathematical modelling, sexually transmitted and blood‐borne infections

## Abstract

**Introduction:**

HIV pre‐exposure prophylaxis (PrEP) has been recommended and partly subsidized in Québec, Canada, since 2013. We evaluated the population‐level impact of PrEP on HIV transmission among men who have sex with men (MSM) in Montréal, Québec's largest city, over 2013–2021.

**Methods:**

We used an agent‐based mathematical model of sexual HIV transmission to estimate the fraction of HIV acquisitions averted by PrEP compared to a counterfactual scenario without PrEP. The model was calibrated to local MSM survey, surveillance, and cohort data and accounted for COVID‐19 pandemic impacts on sexual activity, HIV prevention, and care. PrEP was modelled from 2013 onwards, assuming 86% individual‐level effectiveness. The PrEP eligibility criteria were: any anal sex unprotected by condoms (past 6 months) and either multiple partnerships (past 6 months) or multiple uses of post‐exposure prophylaxis (lifetime). To assess potential optimization strategies, we modelled hypothetical scenarios prioritizing PrEP to MSM with high sexual activity (≥11 anal sex partners annually) or aged ⩽45 years, increasing coverage to levels achieved in Vancouver, Canada (where PrEP is free‐of‐charge), and improving retention.

**Results:**

Over 2013–2021, the estimated annual HIV incidence decreased from 0.4 (90% credible interval [CrI]: 0.3–0.6) to 0.2 (90% CrI: 0.1–0.2) per 100 person‐years. PrEP coverage among HIV‐negative MSM remained low until 2015 (<1%). Afterwards, coverage increased to a maximum of 10% of all HIV‐negative MSM, or about 16% of the 62% PrEP‐eligible HIV‐negative MSM in 2020. Over 2015–2021, PrEP averted an estimated 20% (90% CrI: 11%–30%) of cumulative HIV acquisitions. The hypothetical scenarios modelled showed that, at the same coverage level, prioritizing PrEP to high sexual activity MSM could have averted 30% (90% CrI: 19%–42%) of HIV acquisitions from 2015‐2021. Even larger impacts could have resulted from higher coverage. Under the provincial eligibility criteria, reaching 10% coverage among HIV‐negative MSM in 2015 and 30% in 2019, like attained in Vancouver, could have averted up to 63% (90% CrI: 54%–70%) of HIV acquisitions from 2015 to 2021.

**Conclusions:**

PrEP reduced population‐level HIV transmission among Montréal MSM. However, our study suggests missed prevention opportunities and adds support for public policies that reduce PrEP barriers, financial or otherwise, to MSM at risk of HIV acquisition.

## 1 INTRODUCTION

After over 20 years under study [1, 2, 3] and 10 years of availability [4], oral pre‐exposure prophylaxis (PrEP) has proven highly efficacious for preventing HIV acquisition across transmission routes. Rigorous randomized controlled clinical and pragmatic trials, and other observational data, speak directly to its benefits among men who have sex with men (MSM) when taken daily or on‐demand [5, 6, 7, 8, 9, 10]. The IPERGAY trial in France and Québec (Canada) showed an 86% (95% confidence interval [CI]: 40%–98%) efficacy among MSM assigned to on‐demand oral PrEP compared to placebo [6]. Furthermore, its open‐label extension study suggested that effectiveness could reach 97% (95% CI: 81%–100%) among fully adherent on‐demand PrEP users [8]. However, limited research has examined PrEP's real‐world impact on HIV dynamics over years of implementation, and all existing studies were empirical, relying on observed HIV diagnoses [11, 12], which can be affected by testing efforts.

Individual‐based trials are important to demonstrate individual‐level effectiveness but cannot provide the effect size estimates of public health relevance: the population‐level relative decline in HIV incidence. Beyond the direct benefits to PrEP users, the population‐level effects include the indirect gains accrued by individuals not taking PrEP, who are at reduced risk of HIV acquisition as PrEP interrupts transmission chains and diminishes the number of their contacts able to transmit HIV. However, estimating this population‐level effect empirically can be methodologically challenging, as the ongoing transmission dynamics and use of other prevention tools can make it difficult to define suitable control groups. In Québec, concomitant 2013–2015 changes in antiretroviral treatment (ART) guidelines to immediate initiation and strengthened “undetectable = untransmittable” messaging especially complicate this. Under these circumstances, mathematical modelling can be advantageous [13].

Québec implemented interim guidelines for tenofovir disoproxil fumarate/emtricitabine (TDF‐FTC) as HIV PrEP in 2013 [14], the only province/territory to do so ahead of Health Canada's licencing in 2016 and the development of national PrEP guidelines in 2017 [15]. These guidelines recommended PrEP for MSM who had condomless anal sex in the past 6 months and met one of the following criteria: (1) two or more sex partners in the past 6 months; (2) history of repeated post‐exposure prophylaxis (PEP) use; (3) history of syphilis or an anal bacterial sexually transmitted infection; (4) sex with a partner living with HIV whose risk of transmission is considered high; or (5) psychoactive substance use during sex [14, 16, 17]. Concurrently, Québec's drug insurance programme included TDF‐FTC as PrEP in its formulary, reducing its cost to a monthly co‐payment of up to CAD$97 [18]. However, a decade later, PrEP's impact on the local HIV epidemic has not been evaluated, partly due to the aforementioned challenges. Such evaluations are essential for understanding PrEP's role in eliminating HIV and improving its delivery.

With close to two million people, Montréal is Québec's largest city [19] and its HIV epidemic epicentre [20]. It was the first Canadian city to join the Fast‐Track Cities initiative, aiming to eliminate HIV [21], and has well‐established surveillance and population‐based data for monitoring the HIV response. Leveraging these data, we evaluated the population‐level effectiveness of PrEP on HIV transmission among MSM in Montréal over 2013–2021 and investigated if and how this intervention could have been optimized. Whereas mathematical models have commonly been used to project the potential impacts of PrEP [22, 23, 24, 25], our analysis, informed by real‐world PrEP use data, employed such a tool to retrospectively evaluate PrEP's implementation, disentangling the unique contribution of PrEP from other interventions and simulating an appropriate counterfactual scenario. PrEP is a pillar of HIV elimination efforts [26] and a preferred prevention method for many Canadian MSM [27]. Understanding the impact of PrEP can guide decision‐makers in accelerating the city's progress towards zero new HIV acquisitions.

## 2 METHODS

### 2.1 Model overview

We used an existing calibrated agent‐based model of sexual HIV transmission among Montréal MSM, described elsewhere [28]. Briefly, it is a stochastic, mechanistic model including modules simulating demographics, partnership dynamics (i.e. casual and regular sex partnerships, and mixing by age, serostatus and preferred insertive/receptive role during anal sex), use of HIV prevention (i.e. condoms, PEP, PrEP and viral suppression), HIV testing and ART (Figure S9), HIV transmission and disease progression [28]. Initialized in 1975, the model tracks a population of 10,000 MSM aged 15+ years that increases over time, reflecting Montréal's demographics. Men are categorized by age (15–24, 25–34, 35–44, 45–54, 55+ years) and sexual activity level (low, medium and high, with 0–5, 6–10 and 11+ sexual partners per year, respectively) and exit the model upon death from natural or HIV/AIDS‐related causes. The model is implemented in R (v.4.1.0) with a C++ back‐end using the Rcpp library [29, 30, 31], and simulated with a 2‐week time step.

We calibrated the model to the following outcomes: the distribution of the number of anal sex partners in the past 6 months, prevalence and duration of regular partnerships, CD4 cell count at diagnosis by year (2013–2017), HIV prevalence by age (18–29, 30–49, 50+ years) and year (2005, 2008, 2017–2019), prevalence of lifetime PrEP use by year (2017–2019), PrEP coverage (defined as the proportion currently using PrEP among those not living with HIV) by year (2017–2019), the proportion of people living with HIV (PLHIV) diagnosed by year (2005, 2008, 2017) and ART coverage among PLHIV by year (2005, 2017–2018). Using an Approximate Bayesian Computational Sequential Monte Carlo fitting method [32, 33], we obtained 100 calibrated sets of the 54 parameters governing transmission and interventions coverage that can reproduce the observed epidemic dynamics (Figure S5).

### 2.2 PrEP‐related data sources

We used data from two sources to parameterize and calibrate the PrEP module (Table 1). The *Engage Cohort* [34], a population‐based study of sexually active MSM aged 16+ years in Montréal, Toronto and Vancouver, provided data from 2017 to 2021. In Montréal, a closed cohort of men who had sex with another man in the past 6 months were recruited over 2017–2018 (*N* = 1179) by respondent‐driven sampling (RDS) and visits occurred annually until 2021. The *l'Actuel PrEP Cohort* [35, 36, 37], an ongoing clinical cohort at Montréal's *Clinique l'Actuel*, provided data on individuals consulting for and prescribed PrEP from 2013 to 2019 (*N* = 2746, 98% of which are MSM). All *Engage Cohort* and *l'Actuel PrEP Cohort* participants provided written informed consent prior to data collection.

**Table 1:** Summary of key pre‐exposure prophylaxis (PrEP)‐related model population characteristics, parameters and calibration outcomes

Model population characteristic	Percentage (90% CrI)	Source
**Percentage of MSM not living with HIV eligible for PrEP on 1 January 2013** ^a^	58% (55%–60%)	Model estimate
**Percentage of MSM not living with HIV eligible for PrEP on 1 January 2013 by sexual activity group** ^a^	Low: 31% (28%–33%) Medium: 62% (58%–65%) High: 89% (87%–91%)	Model estimate
**Percentage of MSM not living with HIV eligible for PrEP on 1 January 2013 by age group** ^a^	15–24 years: 44% (42%–46%) 25–54 years: 67% (64%–69%) 55+ years: 48% (44%–52%)	Model estimate
Parameter	**Value or prior distribution**	**Source**
**PrEP effectiveness (assuming the same adherence levels as the source)**	86%	Molina et al. [6]
**Probability of PrEP uptake among MSM eligible for PrEP by year** ^a^	2013–2014: ∼U(0, 0.01) 2015: ∼U(0.01, 0.24) 2016: ∼U(0.01, 0.17) 2017: ∼U(0.01, 0.26) 2018: ∼U(0.06, 0.32) 2019–2022: ∼U(0.06, 0.32)	Calibration (informed by the *Engage Cohort* [34])
**PrEP discontinuation rate (months^−1^)** ^b^	∼U$\left(\frac{1}{13.00},\;\frac{1}{8.44}\right)$	Calibration (informed by *l'Actuel PrEP Cohort* [35]^36^)
**Frequency of HIV testing while on PrEP (months)**	One‐month post‐initiation and every subsequent 3 months	Québec PrEP guidelines [16, 17]
**Probability of attempting to use PEP among MSM eligible for PEP** ^c^	2017: ∼U(0.03, 0.11)	
Calibration outcomes	**Targets**	**Source**
**Prevalence of lifetime PrEP use among all MSM by year**	2017: 6.1%–11.1% 2018: 17.3%–28.0% 2019: 22.5%–36.2%	*Engage Cohort* [34]
**PrEP coverage (i.e. current use) among MSM not living with HIV by year**	2017: 3.1%–6.5% 2018: 7.4%–13.5% 2019: 6.2%–15.9%	*Engage Cohort* [34]

Abbreviations: CrI, credible interval; MSM, men who have sex with men, PEP, post‐exposure prophylaxis; PrEP, pre‐exposure prophylaxis.

^a^ In the model, MSM not living with HIV were eligible for PrEP if they had any anal sex acts unprotected by condoms in the past 6 months and either: (1) ≥2 partnerships in the past 6 months or (2) ≥2 lifetime uses of PEP.

^b^ The PrEP discontinuation rate was converted to a probability for use in a Bernoulli distribution.

^c^ In the model, from 2001 onwards, men not living with HIV who had a casual, condomless anal sex act could attempt to obtain non‐occupational PEP. Beginning at 0% in 2001, the PEP uptake rates increased linearly up to the calibrated 2017 annual rate and were held constant thereafter.

### 2.3 PrEP parameterization

The PrEP module required effectiveness, uptake and discontinuation parameters (Table 1). To parameterize effectiveness, we reviewed published literature and selected the IPERGAY trial's intention‐to‐treat estimate of 86%, considering its relevance to Québec and accounting for imperfect PrEP adherence. We estimated the remaining parameters by calibrating to *Engage* data on lifetime PrEP use among all MSM and PrEP coverage among those not living with HIV.

We calibrated annual probabilities of PrEP uptake among PrEP‐eligible men with prior distributions informed by *Engage*. *Engage* captured self‐reported information on the year of first PrEP use among participants who reported ever using PrEP at baseline and using PrEP in the past 6 months during follow‐up visits. We estimated the proportion of PrEP‐eligible men who first took PrEP each year, accounting for the complex survey design using RDS‐II sampling weights [38] and for loss‐to‐follow‐up using inverse probability of censoring weights (Figures S1 and S2). To obtain estimates before 2017, we assumed the number eligible at baseline was constant. For calibration, we considered the 95% CI bounds and made additional assumptions. First, since no participant reported starting PrEP in 2013 and only seven reported starting in 2014, we assumed uptake was equivalent in those years and informed the prior by the 95% CI of the 2014 estimate. Second, there was increased uncertainty regarding attrition and measurement of PrEP initiation at the last study visit, possibly impacting the estimate for 2019. Since the 95% CI of the 2018 estimate was wide and included plausible values for both years, we used this to inform the prior distribution of the uptake probability in 2019. Finally, we incorporated additional uncertainty in all uptake probability prior distributions to allow for any residual attrition bias (Table 1).

Clinical data from l’*Actuel* informed the PrEP discontinuation rate prior distribution. We defined PrEP discontinuation using three criteria: (1) reported stopping at a follow‐up visit; (2) undergoing another PrEP consultation; or (3) >180 days between visits. The discontinuation date was determined by the available data, as follows: (1) the reported stop date; (2) the date of the visit where stopping was reported; or (3) 3–6 months (randomly chosen from a uniform distribution) after the last visit before stopping. PrEP retention (i.e. duration of continuous use) was calculated as the time between initiation and discontinuation. The prior bounds of the discontinuation rate were obtained by inverting the age‐standardized interquartile range of individual retention (Table 1).

### 2.4 Modelling of PrEP initiation and discontinuation

Starting from 2013, the model simulated oral PrEP use. Matching the Québec guidelines, men susceptible to HIV acquisition were eligible for PrEP if they had any anal sex acts unprotected by condoms in the past 6 months and either: (1) ≥2 partnerships in the past 6 months or (2) ≥2 lifetime PEP uses [16, 17].

At each time step, a Bernoulli distribution using the calibrated uptake probabilities determined if each eligible man would initiate PrEP. Those selected underwent HIV testing and started PrEP if the result was negative. HIV testing while on PrEP occurred after the first month of use and every subsequent 3 months. Those testing positive for HIV immediately discontinued PrEP and started ART.

Finally, the calibrated discontinuation rate was converted to a probability and used in a Bernoulli distribution to determine who discontinued PrEP at each time step.

### 2.5 Impacts of COVID‐19 pandemic disruptions on sexual behaviours and PrEP

The COVID‐19 pandemic's impacts was incorporated into the model starting from March 2020. Sexual activity, prevention, and treatment changes were informed by *Engage* data. PrEP use changes were also informed by *l'Actuel*. From March to June 2020, partner change rates decreased, with a 0.5 and 0.2 absolute reduction in the mean number of annual partners for those living and not living with HIV, respectively, in the low‐medium sexual activity groups and 5.0 and 10.4 partners for those living and not living with HIV, respectively, in the high sexual activity group. Due to service disruptions, reductions in the probabilities of testing annually (by 51% and 21% in the low‐medium and high sexual activity groups, respectively), PEP initiation (by 43%), PrEP initiation (by 35%) and PrEP retention (discontinuation probability increased by 153%) remained until July 2021. After this time, we assumed a return to pre‐pandemic levels.

### 2.6 Model outputs and impact evaluation measures

We tracked characteristics of PrEP use and coverage (percentage of susceptible individuals taking PrEP) and HIV acquisitions over time. We then calculated the annual and cumulative numbers of HIV acquisitions. Finally, we estimated the annual incidence risk by the number of HIV acquisitions each year divided by the number susceptible to HIV acquisition at year‐start.

The HIV epidemic was simulated 10 times for each of the 100 calibrated parameter sets and the outputs were summarized by their mean. We performed this process under two scenarios: the provincial PrEP intervention scenario and the counterfactual scenario without PrEP. We measured PrEP's population‐level impact by the fraction of HIV acquisitions averted (calculated as the number of HIV acquisitions averted by PrEP divided by the total number of acquisitions in the counterfactual scenario without PrEP).

### 2.7 Sensitivity analyses

In sensitivity analyses, we relaxed the eligibility criteria since MSM may have received PrEP despite not meeting all the provincial criteria [39]. We considered two scenarios, each applying only one eligibility criterion at a time: (1) condomless anal sex in the past 6 months, and (2) ≥2 partnerships in the past 6 months. The PrEP uptake rates were kept as calibrated.

Additionally, we assessed the robustness of our results to the assumed PrEP effectiveness, increasing it to 96%, as data indicated high adherence among continuous users (Supplementary Materials).

### 2.8 Alternative PrEP intervention scenarios

We simulated alternative, hypothetical intervention scenarios to explore how PrEP's impact could have been optimized (Table 2). These involved assessing the potential impact of PrEP prioritization (either to men in the high sexual activity group or those aged ⩽45 years), increased coverage levels (up to a maximum of 30% by 2019, approximating the coverage reached in Vancouver, where PrEP is free for eligible individuals) and increased retention (reducing the discontinuation probability by 25% and 50%).

**Table 2:** Pre‐exposure prophylaxis (PrEP) intervention scenarios modelled among men who have sex with men (MSM) in Montréal over 2013–2021

Scenario	PrEP eligibility criteria	PrEP usage
***Provincial PrEP intervention scenario***		
Provincial PrEP intervention	Provincial criteria^a^	Applied calibrated uptake rates
***Alternative PrEP intervention scenarios***		
Prioritized high‐activity users	High sexual activity group	Matched the coverage of the provincial intervention scenario over time
Prioritized younger users	Aged ⩽45 years	Matched the coverage of the provincial intervention scenario over time
Increased coverage	Provincial criteria^a^	Matched coverage targets of 5% or 10% by 2015 and 20% or 30% by 2019^b^
Increased coverage + prioritized high‐activity users	High sexual activity group	Matched coverage targets of 5% or 10% by 2015 and 20% or 30% by 2019^b^
Increased coverage + prioritized younger users	Aged ⩽45 years	Matched coverage targets of 5% or 10% by 2015 and 20% or 30% by 2019^b^
Increased retention (probability of discontinuation reduced by 25% and 50%)	Provincial criteria^a^	Applied calibrated uptake rates

Abbreviation: MSM, men who have sex with men; PrEP, pre‐exposure prophylaxis.

^a^ In the model, MSM not living with HIV were eligible for PrEP if they had any anal sex acts unprotected by condoms in the past 6 months and either: (1) ≥2 partnerships in the past 6 months or (2) ≥2 lifetime uses of post‐exposure prophylaxis.

^b^ Starting with 0% coverage in 2013 and using linear interpolation to set the targeted coverage between 2013–2015 and 2015–2019. The 2019 coverage target was then maintained through to the end of 2021.

### 2.9 Ethics

The *McGill University Research Ethics Board* and the *Research Institute of the McGill University Health Centre* approved this study.

## 3 RESULTS

### 3.1 PrEP coverage

**Figure 1:**
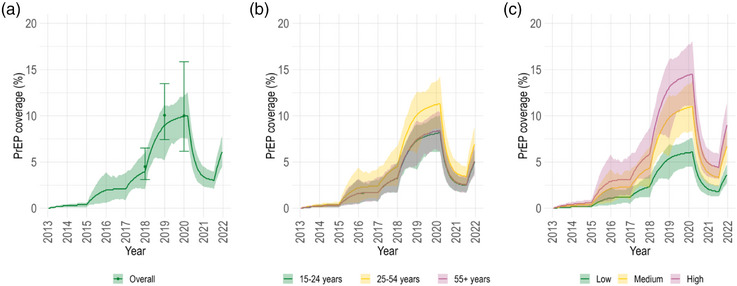
Pre‐exposure prophylaxis (PrEP) coverage among men who have sex with men (MSM) not living with HIV in Montréal. Estimated PrEP coverage over 2013–2021 among MSM not living with HIV in Montréal: overall (panel A) and stratified by age (panel B) and sexual activity group (panel C). The coloured lines and bands show the model posterior mean and 90% credible intervals, respectively. The three points and bars in panel A display the estimated PrEP coverage and 95% confidence intervals calculated from *Engage* and adjusted by RDS‐II and inverse probability of censoring weights.

According to *Engage* data, PrEP uptake was low after Québec's interim guidelines were published in 2013 and gradually started increasing in 2015, reaching 5% (95% CI: 3%–7%) coverage among MSM not living with HIV by 2018 (Figure 1). In 2020, *Engage* data indicated that 10% (95% CI: 6%–16%) of Montréal MSM not living with HIV were currently on PrEP. Our model reflected these trends well, matching the estimated PrEP coverage at 4% (90% credible interval [CrI]: 2%–6%) in 2018 and 10% (90% CrI: 8%–12%) in 2020 (Figure 1). Among the PrEP‐eligible population, which accounted for 62% (90% CrI: 60%–65%) of MSM not living with HIV (Figure S6), current use reached 16% (90% CrI: 12%–20%) in 2020 (Figure S7). During the initial waves of the COVID‐19 pandemic, there was a large decline in PrEP coverage due to reduced initiation and increased discontinuation (by model design), but coverage rebounded in mid‐2021 (Figure 1). Throughout the study period, PrEP usage varied across different age and sexual activity groups (Figure 1).

### 3.2 Annual HIV incidence

**Figure 2:**
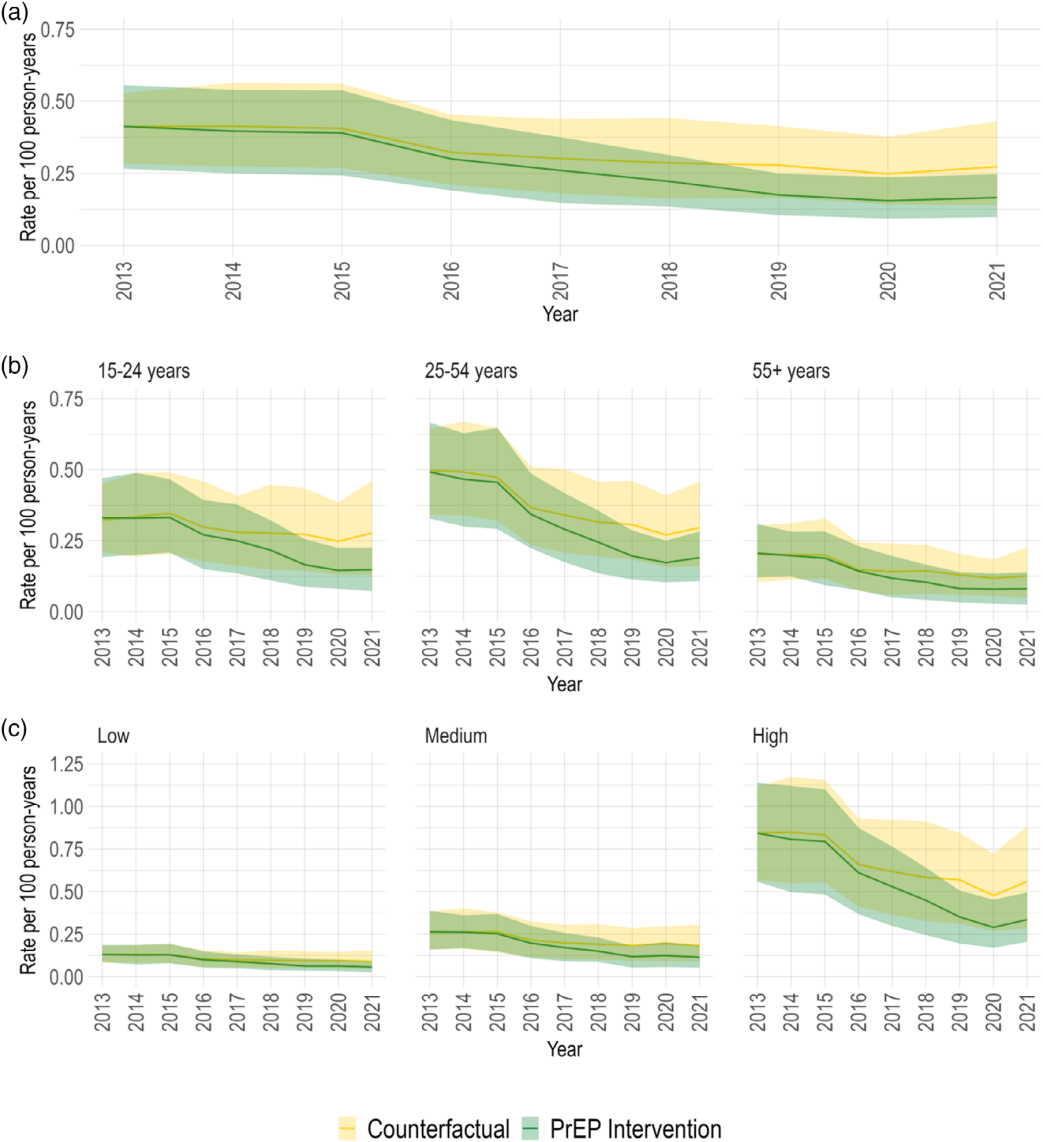
Annual HIV incidence in the provincial pre‐exposure prophylaxis (PrEP) intervention and counterfactual scenarios. Estimated HIV incidence rates over 2013–2021 among men who have sex with men (MSM) in Montréal under the provincial PrEP intervention and counterfactual scenarios. The rates are presented overall (panel A) and stratified by age (panel B) and sexual activity group (panel C). The coloured lines and bands show the posterior mean and 95% credible intervals, respectively.

**Figure 3:**
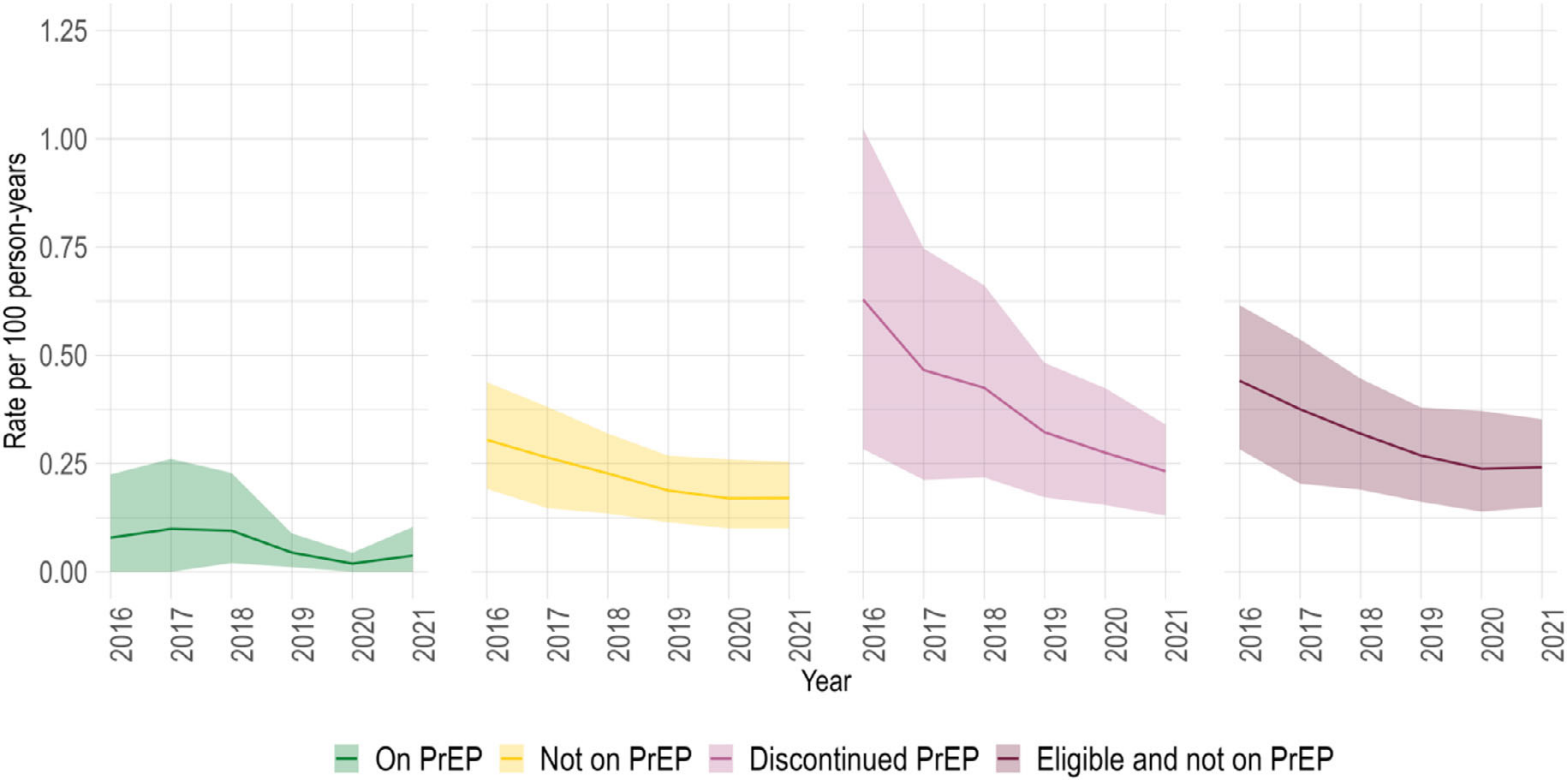
Annual HIV incidence by pre‐exposure (PrEP) use status in the provincial PrEP intervention scenario. Estimated HIV incidence rates under the provincial pre‐exposure prophylaxis (PrEP) intervention scenario over 2013–2021 among men who have sex with men (MSM) in Montréal, stratified by PrEP use status. The coloured lines and bands show the posterior mean and 95% credible intervals, respectively.

The modelled annual HIV incidence was 0.4 per 100 person‐years (90% CrI: 0.3–0.6) in 2013 and decreased to 0.2 per 100 person‐years (90% CrI: 0.1–0.2) in 2021 (Figure 2). Prior to the PrEP scale‐up, the estimated annual incidence differed markedly by age and sexual activity levels (Figure 2). In 2013, the oldest age group (55+) had the lowest estimated annual incidence (0.2 per 100 person‐years [90% CrI: 0.1–0.3]) compared to the 15‐ to 24‐year‐olds (0.3 per 100 person‐years [90% CrI: 0.2–0.5]) and the 25‐ to 54‐year‐olds (0.5 per 100 person‐years [90% CrI: 0.3–0.7]). By the end of 2020, incidence reached 0.1 per 100 person‐years in the 15–24 (90% CrI: 0.1–0.2) and 55+ (90% CrI: 0–0.1) age groups, and 0.2 per 100 person‐years (90% CrI: 0.1–0.3) in those aged 25–54. Across sexual activity levels, those with more sexual partners had a higher incidence. Over time, the most pronounced incidence reductions were exhibited by the highest sexual activity group, decreasing from 0.8 per 100 person‐years (90% CrI: 0.6–1.1) in 2013 to 0.3 per 100 person‐years (90% CrI: 0.2–0.5) in 2021. Considering PrEP use status over 2016–2021, the incidence was higher among MSM eligible but not taking PrEP and even higher among those who discontinued PrEP (Figure 3).

### 3.3 Impact evaluation

**Figure 4:**
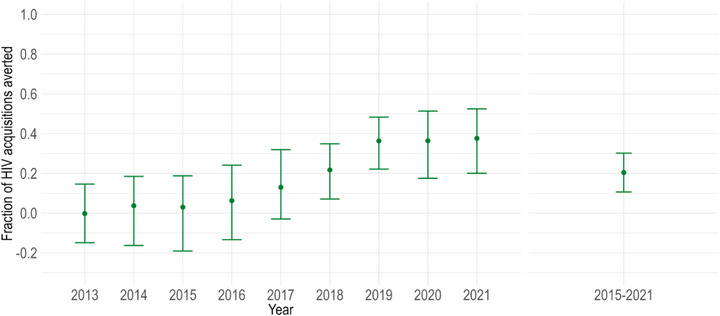
HIV acquisitions averted under the provincial pre‐exposure prophylaxis (PrEP) intervention scenario. Estimated annual (2013–2021) and cumulative (2015–2021) fractions of acquisitions averted due to the provincial PrEP intervention among men who have sex with men in Montréal. The coloured points and bars show the posterior mean and 90% credible intervals, respectively.

Over the study period, the annual fractions of HIV acquisitions averted by PrEP increased (Figure 4). In the early years of PrEP availability, when coverage was lowest, it had a limited impact on averting HIV acquisitions. However, starting in 2017, PrEP began to have greater impacts. In 2021, PrEP averted an estimated 38% (90% CrI: 20%–53%) of HIV acquisitions. Given the low coverage before 2015, we focused on the cumulative evaluation from 2015 onwards and estimated that PrEP averted 20% (90% CrI: 11%–30%) of HIV acquisitions from 2015 to the end of 2021 (Table 3 and Figure 4).

**Table 3:** Cumulative pre‐exposure prophylaxis (PrEP) impact evaluation results among men who have sex with men (MSM) in Montréal over 2015–2021

Scenario	Number of HIV acquisitions averted in the total MSM population^a^ *N* (90% CrI)	Fraction of HIV acquisitions averted % (90% CrI)	Number of person‐years on PrEP needed to prevent one HIV acquisition^b^ Person‐years (90% CrI)
***Provincial PrEP intervention scenario***			
Provincial PrEP intervention	207 (81–365)^c^	20% (11%–30%)	106 (40–172)
***Alternative PrEP intervention scenarios***			
Prioritized high‐activity users	302 (144–522)	30% (19%–42%)	63 (31–106)
Prioritized younger users	176 (59–324)	18% (8%–28%)	117 (47–251)
Increased coverage:			
5% by 2015–20% by 2019	482 (288–738)	49% (39%–57%)	108 (6–168)
5% by 2015–30% by 2019	554 (338–855)	56% (48%–63%)	135 (79–203)
10% by 2015–20% by 2019	563 (320–837)	57% (46%–65%)	104 (63–168)
10% by 2015–30% by 2019	621 (338–954)	63% (54%–70%)	130 (77–219)
Increased coverage + prioritized high‐activity users^d^:			
5% by 2015–20% by 2019	608 (347–932)	61% (52%–68%)	86 (51–140)
10% by 2015–20% by 2019	684 (410‐1040)	70% (62%–76%)	85 (51–134)
Increased coverage + prioritized younger users:			
5% by 2015–20% by 2019	414 (239–626)	42% (32%–50%)	128 (74–200)
5% by 2015–30% by 2019	491 (266–783)	50% (42%–58%)	154 (86–260)
10% by 2015–20% by 2019	495 (279–761)	50% (41%–58%)	119 (70–192)
10% by 2015–30% by 2019	554 (324–837)	56% (47%–63%)	147 (88–234)
Increased retention:			
probability of discontinuation reduced by 25%	279 (140–500)	28% (18%–41%)	149 (77–269)
probability of discontinuation reduced by 50%	234 (99–419)	23% (14%–33%)	139 (61–251)

Abbreviations: MSM, men who have sex with men; PrEP, pre‐exposure prophylaxis.

^a^ Assuming the model population represents 22% of the total MSM population in Montréal (see Table S1 for estimates of the MSM population size over 2015–2021).

^b^ Calculated as the total person years on PrEP divided by the number of HIV acquisitions averted.

^c^ Annual numbers of HIV acquisitions averted under the provincial PrEP intervention scaled to the total MSM population are presented in Table S1.

^d^ It was not possible to reach 30% coverage by 2019 due to the insufficient number of individuals in the high sexual activity group.

### 3.4 Sensitivity analyses

Our estimation of HIV incidence under PrEP intervention was not sensitive to the level of PrEP efficacy (86% vs. 96%) or loosened eligibility criteria (Figure S10).

### 3.5 Alternative PrEP intervention scenarios

**Figure 5:**
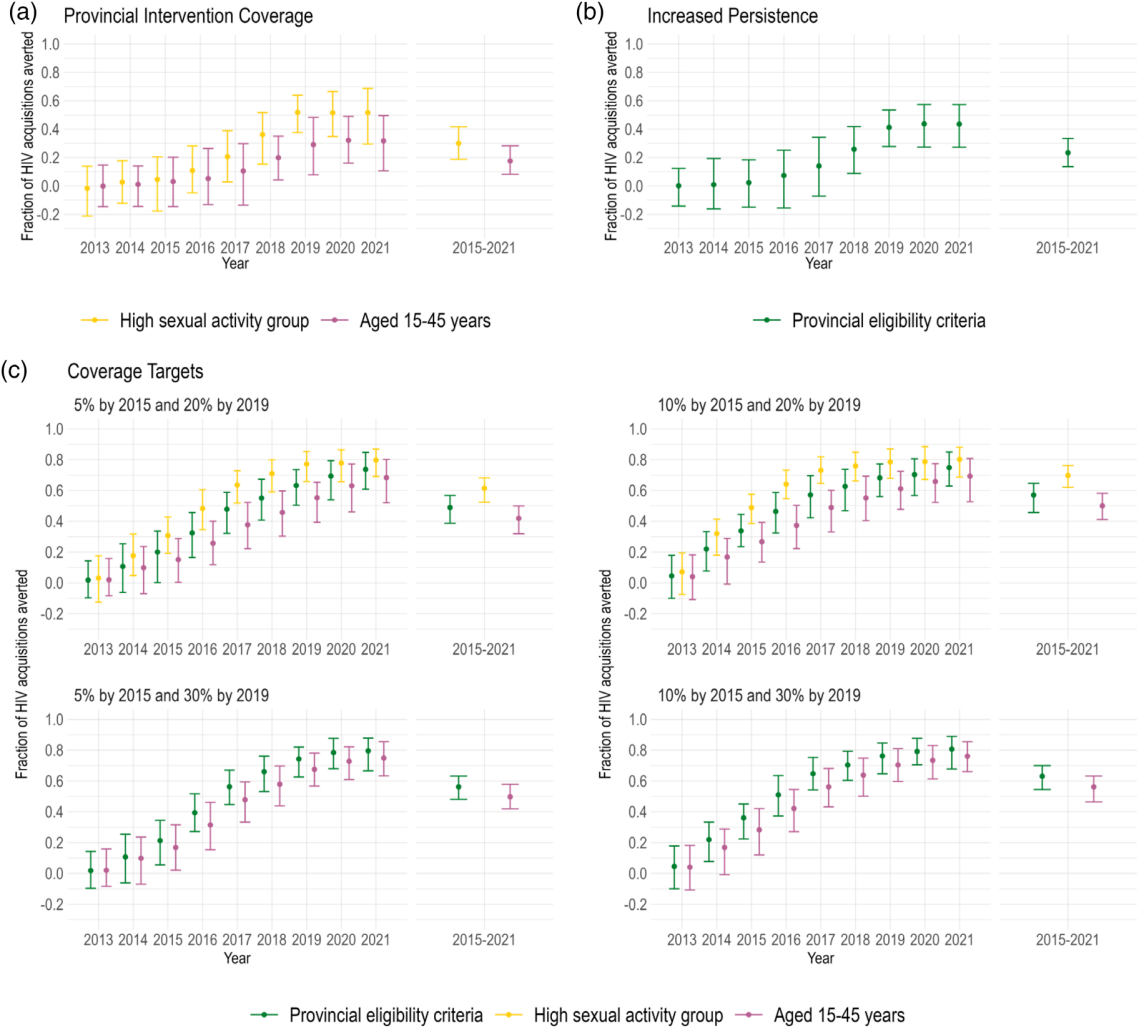
Estimated fraction of acquisitions averted due to pre‐exposure prophylaxis (PrEP) intervention among men who have sex with men (MSM) in Montréal under alternative (hypothetical) intervention scenarios. For each, the annual estimates from 2013 to 2021 and cumulative estimates over 2015–2021 are shown. The coloured points and bars show the posterior mean and 95% credible intervals, respectively. The panels display the results of: (A) maintaining the observed PrEP coverage but prioritizing uptake in (1) the high sexual activity group or (2) those aged 15–45 years; (B) maintaining the calibrated PrEP uptake probabilities but increasing retention on PrEP (for simplicity, only the results of the 50% decrease in discontinuation probability scenario are plotted); and (C) increasing coverage up to a maximum of 30% by 2019 for three different uptake assumptions: (1) the same as the provincial PrEP eligibility criteria, or prioritizing uptake in (2) the high sexual activity group or (3) those aged 15–45 years. Note that, in the scenarios prioritizing MSM in the high sexual activity group, it was not possible to reach 30% coverage by 2019 due to the insufficient number of individuals in the group. The results for these scenarios are not presented.

Our alternative (hypothetical) analyses (Table 2) suggested that, to have improved the impact of PrEP compared to the provincial intervention scenario, prioritizing MSM in the high sexual activity group (same overall coverage) or attaining higher overall PrEP coverage of 5% or 10% and 20% or 30% coverage by 2015 and 2019, respectively, would have been needed (Table 3 and Figure 5). Prioritizing PrEP to MSM in the high sexual activity group (same coverage as the provincial intervention) cumulatively averted 30% (90% CrI: 19%–42%) of HIV acquisitions over 2015–2021, with a peak annual fraction averted of 52% (90% CrI: 30%–69%) in 2021 (Table 3 and Figure 5). Even higher impacts could have have been acheived by reaching coverage targets of 5% or 10% by 2015 and 20% or 30% by 2019, especially when combined with prioritized use for MSM with high sexual activity levels. For instance, the scenario with the smallest increase in PrEP coverage, reaching targets of 5% in 2015 and 20% in 2019, averted 49% (90% CrI: 39%–57%) of HIV acquisitions between 2015 and 2022 (under provincial eligibility criteria), and up to 68% (90% CrI: 54%–70%) when prioritizing PrEP to the high sexual activity group. Conversely, the scenario with the largest increase in PrEP coverage, mirroring the coverage in Vancouver, reached targets of 10% by 2015 and 30% by 2019 and, under provincial eligibility criteria, averted 63% (90% CrI: 54%–70%) of HIV acquisitions over 2015–2021.

## 4 DISCUSSION

This study presents a population‐level estimate of PrEP's impact, considering both PrEP's direct and indirect effects. Using a detailed agent‐based model of sexual HIV transmission and prevention among Montréal's MSM, we created a counterfactual scenario without PrEP and found that, despite relatively low coverage, PrEP may have averted 20% (90% CrI: 11%–30%) of new HIV acquisitions between 2015 and the end of 2021 in this population. From 2015 to 2019, as time and coverage accrued to 10% of MSM not living with HIV and 16% of the PrEP‐eligible, the annual fraction of acquisitions averted by PrEP rose from 3% to 36%. Afterwards, despite the COVID‐19 pandemic disruptions to coverage, this level of impact persisted.

Although our analysis suggests considerable population‐level benefits of PrEP intervention among MSM in Montréal, it also highlights missed prevention opportunities. One way to improve PrEP's impact is to attain higher coverage. At the observed levels, prioritizing MSM with higher sexual activity levels might have improved impact, but not to the same extent as achieving higher coverage with the current provincial eligibility criteria, which approximately 60% of MSM not living with HIV met in our model. Increasing coverage to 5% or 10% in 2015 and 20% or 30% in 2019 could have prevented more than twice the number of HIV acquisitions since 2015. Given the coverage estimates over 2017–2020 for Vancouver MSM, we believe that levels of 10% in 2015 and 30% in 2019 could have been feasible in Montréal and an estimated 63% (90% CrI: 54%–70%) of HIV acquisitions would have been averted instead of 20%. Vancouver's higher coverage has been attributed to total public funding for PrEP in that province [40], whereas the PrEP co‐payment in Québec can be as high as CAD$97 per month [18]. Even matching 5% coverage in 2015 and 20% in 2019 could have averted 49% (90% CrI: 39%–57%) of HIV acquisitions between 2015 and 2022, rising to 68% (90% CrI: 54%–70%) if efforts focused on MSM frequently engaging in anal sex with different partners.

Two other studies have examined the impact of PrEP intervention empirically using surveillance data and observed changes in HIV diagnoses pre‐ and post‐PrEP implementation [11, 12]. In Australia's state of New South Wales, PrEP reached an estimated 20% of MSM living without HIV in 2016 and reduced new diagnoses of recent HIV acquisitions among MSM by 31.5% (95% CI: 11.3%–47.3%) in a 12‐month post‐implementation period, compared to the year prior [11]. A similar study in Scotland, where PrEP is delivered free‐of‐cost from national clinics, estimated that 20% of MSM attending such clinics were prescribed PrEP and showed a 35.6% (95% CI: 7.1%–55.4%) reduction in new diagnoses of recent HIV acquisitions among MSM over a 24‐month post‐implementation period [12].

It is challenging to directly compare these findings to ours due to different time frames (PrEP's impact is expected to accrue over time) and variations in coverage and use. The most comparable period of our study is over 2017 and 2018 when coverage was beginning to rise. In those years, we estimated that 13% (90% CrI: 0%–32%) and 22% (90% CrI: 7%–35%) of acquisitions were averted, respectively. While differences between our estimates may stem from lower coverage in our study, heterogeneity in risk across the populations could also differ. Additionally, other factors like changes in HIV testing trends, the use of diagnoses of recent acquisitions to proxy incidence, and concomitant improvements in the treatment and care cascade could influence the findings of these studies.

Our analysis helped highlight missed opportunities in PrEP delivery. For example, it took approximately 2 years for PrEP coverage to start increasing after Québec issued guidelines. Increased awareness of PrEP's efficacy and safety among healthcare providers and potential users following the early cessation of the IPERGAY trial in 2014 could have encouraged earlier use. Even after PrEP expanded, coverage plateaued at 10%, never reaching Vancouver's levels. Our model indicated that many eligible MSM were not engaged in PrEP care yet could have benefited from its use, as evidenced by their higher HIV incidence. The financial burden of PrEP in Montréal remains an important barrier, potentially leaving behind those most at risk of HIV acquisition [41].

The findings from our impact evaluation should be interpreted cautiously. Our model used simplifying assumptions about disease progression, HIV transmission and prevention use. For instance, individuals on PrEP may be less likely to serosort (i.e. choose partners not living with HIV), and PLHIV may preferentially mix with PrEP users [34, 42]. If mixing was less assortative by HIV status among those on PrEP, we might have slightly overestimated the impact of PrEP. However, this potential bias should be small given the high viral suppression levels observed in Montréal. Additionally, the model did not consider risk compensation or changes in sexual behaviour that could be associated with PrEP use, which could overestimate the impact of PrEP. However, this overestimation should be small, given PrEP's high effectivness. Finally, the model did not differentiate between daily and on‐demand regimens, given their equivalent efficacy under perfect adherence.

Our analysis has several strengths. Firstly, we leveraged data from multiple cohorts and surveys of MSM in Montréal to parameterize and calibrate the model. Secondly, using a model allowed us to construct an appropriate counterfactual scenario when there was no other control group available [13]. Thirdly, we controlled for changes in Québec's ART eligibility criteria. Lastly, our impact estimates include PrEP's direct effects in preventing HIV acquisition among users and the indirect benefits to MSM not on PrEP due to overall decreases in HIV prevalence.

## 5 CONCLUSIONS

PrEP has the potential to contribute significantly to HIV elimination as a key part of combination HIV prevention. However, global scale‐up has been slow, resulting in sub‐optimal coverage and limited impact on incidence reductions [43]. In Montréal, despite modest coverage, PrEP had a notable impact on HIV transmission, complementing declining incidence and high ART coverage. Free PrEP could remove important barriers, but more needs to be done to address stigma, discrimination and certain physicians’ reticence to prescribe PrEP, among others [44, 45, 46, 47, 48, 49, 50]. The availability of alternative formulations like long‐acting injectable PrEP could further usage and support adherence [50]. By removing barriers, we can accelerate HIV elimination among MSM and other vulnerable populations [51].

## COMPETING INTERESTS

JC has investigator‐sponsored research grants from Gilead Sciences Canada and ViiV Healthcare. He has also received financial support for conference travel and advisory work for Gilead Sciences Canada, Merck Canada and ViiV Healthcare. MM‐G reports an investigator‐sponsored research grant from *Gilead Sciences Inc*., contractual arrangements from the *World Health Organization*, the *Joint United Nations Programme on HIV/AIDS* (UNAIDS), the *Institut national de santé publique du Québec* (INSPQ) and *the Institut d'excellence en santé et services sociaux* (INESSS), all outside of the submitted work. CT has investigator‐sponsored research grants from Merck and Gilead Sciences Canada, and has received financial support for advisory work and conferences from Gilead Sciences Canada, Merck, Medicago, Astra‐Zeneca, Pfizer, Sanofi and GSK. J‐GB has received honoraria for consulting for ViiV, Healthcare, Merck and Gilead Sciences Canada and for participation as a speaker at conferences supported by Merck and Gilead Sciences unrelated to this work.

## AUTHORS’ CONTRIBUTIONS

CMD, RMM, MM‐G and JC contributed to the study conception and design. RMM, CMD, YX and MM‐G contributed to model development, parameterization and calibration. JC, GL, DMM, DG and RT were involved in the data collection. Analyses were performed by CMD, with support from MM‐G, JC and RMM. The manuscript was drafted by CMD. All authors contributed to the interpretation of results and reviewed the manuscript for important intellectual content. Overall supervision for this project was provided by MM‐G and JC. All authors approved the final manuscript.

## FUNDING

CMD is supported by a doctoral award from the *Fonds de recherche du Québec—Santé* (FRQS). Grants from the *Canadian Foundation for AIDS Research* and the *Canadian Institutes of Health Research* (CIHR) to MM‐G. MM‐G's research programme is funded by the *Tier 2 Canada Research Chair* in *Population Health Modelling*. SM is supported the *Tier 2 Canada Research Chair* in *Mathematical Modelling and Program Science* and the *Ontario HIV Treatment Network*. CT is the *Pfizer/Université de Montréal Chair* in *HIV Translational Research*.

MCB acknowledges funding from the *MRC Centre for Global Infectious Disease Analysis* (reference MR/R015600/1), jointly funded by the *UK Medical Research Council* (MRC) and the *UK Foreign, Commonwealth & Development Office* (FCDO), under the MRC/FCDO Concordat agreement and is also part of the EDCTP2 programme supported by the European Union. For the purpose of open access, MCB has applied a Creative Commons Attribution (CC BY) license to any Author Accepted Manuscript version arising. DG is supported by the *Tier 2 Canada Research Chair* in *Sexual and Gender Minority Health Research*.

## DISCLOSURES

RÉZO (AD‐B) has received sponsorships from Gilead Sciences Canada.

## DISCLAIMER

This work is the sole product of the authors and has never been submitted for publication. A pre‐print of this manuscript is available online at https://www.medrxiv.org/content/10.1101/2023.05.31.23290795v1.full.

## Supporting information


**Figure S1. Empirical estimates of pre‐exposure prophylaxis (PrEP) eligibility among men who have sex with men (MSM) in Montréal**. The estimated annual percentage of Montréal MSM eligible for PrEP (according to the provincial criteria) among Engage participants that self‐reported a negative or unknown HIV serostatus. The first four Engage study visits occurred annually over 2017‐2021. All estimates were adjusted by RDS‐II and inverse probability of censoring weights. The error bars show the estimated 95% confidence intervals.
**Figure S2. Empirical estimates of pre‐exposure prophylaxis (PrEP) uptake among men who have sex with men (MSM) in Montréal**. The estimated percentage of Montréal MSM that reported first taking PrEP each year among Engage participants eligible for PrEP (according to the modelled criteria). No Engage participant reported first taking PrEP in 2013. To obtain estimates before 2017 (when the study began), we assumed the number eligible at baseline was constant. All estimates were adjusted by RDS‐II and inverse probability of censoring weights. The error bars show the estimated 95% confidence intervals.
**Figure S3. Empirical estimates of pre‐exposure prophylaxis (PrEP) adherence among men who have sex with men (MSM) in Montréal**. Estimates of PrEP adherence among Montréal MSM calculated using the Engage cohort and adjusted by RDS‐II and inverse probability of censoring weights. The first four Engage study visits occurred annually over 2017‐2021. Panel A displays the self‐reported average number of pills missed per week among continuous PrEP users at each study visit. Panel B displays the self‐reported percentage of anal sex acts covered by PrEP among continuous PrEP users at the third and fourth study visits. The error bars show the estimated 95% confidence intervals.
**Figure S4. Empirical estimates of pre‐exposure prophylaxis (PrEP) dosing schedule among men who have sex with men (MSM) in Montréal**. The estimated percentage of PrEP users following a daily or on‐demand dose schedule calculated using the Engage cohort and adjusted by RDS‐II and inverse probability of censoring weights. The error bars show the estimated 95% confidence intervals. The first four Engage study visits occurred annually over 2017‐2021.
**Figure S5. Select model calibration results reproduced from Milwid et al^8^**. Model calibration produced 100 parameter sets, each of which were simulated once to produce figures of the HIV prevalence over 1975‐2019 (panel A), the proportion of new HIV diagnoses in each CD4 cell count category over 2013‐2017 (panel B), the proportion of diagnosed people living with HIV on antiretroviral treatment (ART) in 2005, 2017, and 2018 (panel C), and the proportion that ever used pre‐exposure prophylaxis (PrEP) over 2017‐2019. The purple lines boxplots show the model simulations. The orange points and error bars show the target data used in calibration. For results of the remaining calibration outcomes, please refer to the supplementary materials of our previous model publication by Milwid et al^8^.
**Figure S6. Modelled pre‐exposure prophylaxis (PrEP) eligibility among men who have sex with men (MSM) not living with HIV in Montréal**. The model estimated percentage of MSM not living with HIV eligible for PrEP over 2013‐2021 in Montréal: overall (panel A) and stratified by age (panel B) and sexual activity group (panel C). The coloured lines and bands show the model posterior mean and 90% credible intervals, respectively.
**Figure S7. Modelled pre‐exposure prophylaxis (PrEP) coverage among PrEP‐eligible men who have sex with men (MSM) not living with HIV in Montréal**. The model estimated PrEP coverage over 2013‐2021 among MSM eligible for PrEP in Montréal: overall (panel A) and stratified by age (panel B) and sexual activity group (panel C). The coloured lines and bands show the model posterior mean and 90% credible intervals, respectively.
**Figure S8. Annual HIV incidence across ten simulations of one parameter set**. Estimated HIV incidence rates over 2013‐2021 among men who have sex with men (MSM) in Montréal under the provincial pre‐exposure prophylaxis (PrEP) intervention scenario. The green lines display the results per simulation and the black line displays the median.
**Figure S9. Modelled antiretroviral treatment (ART) and viral load suppression coverage among men who have sex with men (MSM) living with HIV (PLHIV) in Montréal**. The model ART and viral load suppression coverage among MSM living with HIV over 2013‐2021. From 2013 onward, all PLHIV in the model are eligible for ART and initiate upon HIV diagnosis. The coloured lines and bands show the model posterior mean and 90% credible intervals, respectively. The two points and bars display the estimated ART coverage and 95% confidence intervals calculated from Engage and adjusted by RDS‐II and inverse probability of censoring weights.
**Figure S10. Sensitivity Analyses**. The model estimated HIV incidence rates over 2013‐2021 among Montréal men who have sex with men (MSM) under the provincial pre‐exposure prophylaxis (PrEP) intervention scenario with different PrEP‐eligibility criteria (panel A) and with different PrEP efficacies (Panel B). The coloured lines and bands show the posterior median and 90% credible intervals, respectively.
**Figure S11. Cumulative Acquisitions Averted**. Estimated cumulative fraction of acquisitions averted due to pre‐exposure prophylaxis (PrEP) intervention among men who have sex with men (MSM) in Montréal (provincial PrEP intervention scenario) over varying time periods. The coloured points and bars show the posterior mean and 90% credible intervals, respectively.
**Table S1**. Annual pre‐exposure prophylaxis (PrEP) impact evaluation results among men who have sex with men in Montréal over 2015‐2021 in the model population and scaled to the total population.

## Data Availability

The data that support the findings of this study are available from Engage. These data are not publicly available and restrictions do apply for access. Engage permitted the use of these data for this study. The data could be available from the authors upon reasonable request and with permission of Engage.

## References

[1] Tsai C‐C , Follis KE , Sabo A , Beck TW , Grant RF , Bischofberger N , et al. Prevention of SIV infection in macaques by (*R*)‐9‐(2‐phosphonylmethoxypropyl)adenine. Science. 1995; 270(5239):1197–1199. 7502044 10.1126/science.270.5239.1197

[2] García‐Lerma JG , Otten RA , Qari SH , Jackson E , Cong M‐E , Masciotra S , et al. Prevention of rectal SHIV transmission in macaques by daily or intermittent prophylaxis with emtricitabine and tenofovir. PLoS Med. 2008; 5(2):e28. 18254653 10.1371/journal.pmed.0050028 PMC2225435

[3] Denton PW , Estes JD , Sun Z , Othieno FA , Wei BL , Wege AK , et al. Antiretroviral pre‐exposure prophylaxis prevents vaginal transmission of HIV‐1 in humanized BLT mice. PLoS Med. 2008; 5(1):e16. 18198941 10.1371/journal.pmed.0050016 PMC2194746

[4] CDC statement on FDA approval of drug for HIV prevention [press release]. July 16 2012.

[5] Grant RM , Lama JR , Anderson PL , McMahan V , Liu AY , Vargas L , et al. Preexposure chemoprophylaxis for HIV prevention in men who have sex with men. N Engl J Med. 2010; 363(27):2587–2599. 21091279 10.1056/NEJMoa1011205 PMC3079639

[6] Molina JM , Capitant C , Spire B , Pialoux G , Cotte L , Charreau I , et al. On‐demand preexposure prophylaxis in men at high risk for HIV‐1 infection. N Engl J Med. 2015; 373(23):2237–2246. 26624850 10.1056/NEJMoa1506273

[7] McCormack S , Dunn DT , Desai M , Dolling DI , Gafos M , Gilson R , et al. Pre‐exposure prophylaxis to prevent the acquisition of HIV‐1 infection (PROUD): effectiveness results from the pilot phase of a pragmatic open‐label randomised trial. Lancet. 2016; 387(10013):53–60. 26364263 10.1016/S0140-6736(15)00056-2 PMC4700047

[8] Molina JM , Charreau I , Spire B , Cotte L , Chas J , Capitant C , et al. Efficacy, safety, and effect on sexual behaviour of on‐demand pre‐exposure prophylaxis for HIV in men who have sex with men: an observational cohort study. Lancet HIV. 2017; 4(9):e402–e410. 28747274 10.1016/S2352-3018(17)30089-9

[9] Huang X , Hou J , Song A , Liu X , Yang X , Xu J , et al. Efficacy and safety of oral TDF‐based pre‐exposure prophylaxis for men who have sex with men: a systematic review and meta‐analysis. Front Pharmacol. 2018; 9:799. 30233355 10.3389/fphar.2018.00799 PMC6131617

[10] Antoni G , Tremblay C , Delaugerre C , Charreau I , Cua E , Rojas Castro D , et al. On‐demand pre‐exposure prophylaxis with tenofovir disoproxil fumarate plus emtricitabine among men who have sex with men with less frequent sexual intercourse: a post‐hoc analysis of the ANRS IPERGAY trial. Lancet HIV. 2020; 7(2):e113–e120. 31784343 10.1016/S2352-3018(19)30341-8

[11] Grulich AE , Guy R , Amin J , Jin F , Selvey C , Holden J , et al. Population‐level effectiveness of rapid, targeted, high‐coverage roll‐out of HIV pre‐exposure prophylaxis in men who have sex with men: the EPIC‐NSW prospective cohort study. Lancet HIV. 2018; 5(11):e629–e637. 30343026 10.1016/S2352-3018(18)30215-7

[12] Estcourt C , Yeung A , Nandwani R , Goldberg D , Cullen B , Steedman N , et al. Population‐level effectiveness of a national HIV preexposure prophylaxis programme in MSM. AIDS. 2021; 35(4):665–673. 33290298 10.1097/QAD.0000000000002790 PMC7924973

[13] Boily MC , Lowndes CM , Vickerman P , Kumaranayake L , Blanchard J , Moses S , et al. Evaluating large‐scale HIV prevention interventions: study design for an integrated mathematical modelling approach. Sex Transm Infect. 2007; 83(7):582–589. 17942574 10.1136/sti.2007.027516 PMC2598645

[14] Ministère de la Santé et des Services sociaux . Avis intérimaire sur la prophylaxie préexposition au virus de l'immunodéficience humaine. Gouvernment du Quebec; 2013.

[15] Tan DHS , Hull MW , Yoong D , Tremblay C , O'Byrne P , Thomas R , et al. Canadian guideline on HIV pre‐exposure prophylaxis and nonoccupational postexposure prophylaxis. Can Med Assoc J. 2017; 189(47):E1448–E1458. 29180384 10.1503/cmaj.170494 PMC5703677

[16] Ministère de la Santé et des Services sociaux . La prophylaxie préexposition au virus de l'immunodéficience humaine: guide pour les professionnels de la santé du Québec. Gouvernement du Québec; 2017.

[17] Ministère de la Santé et des Services sociaux . La prophylaxie préexposition au virus de l'immunodéficience humaine: guide pour les professionnels de la santé du Québec. Gouvernement du Québec; 2019.

[18] Régie de l'assurance maladie du Québec . Tarifs en vigueur. Gouvernement du Québec; 2023.

[19] Statistics Canada . Table 17‐10‐0005‐01 Population estimates on July 1st, by age and sex [Internet]. 2021 [cited 2022]. Available from: https://www150.statcan.gc.ca/t1/tbl1/en/tv.action?pid=1710000501. Accessed 15 June 2021.

[20] Doyle CM , Cox J , Milwid RM , Bitera R , Delaunay CL , Alary M , et al. Measuring progress towards reaching zero new HIV acquisitions among key populations in Québec (Canada) using routine surveillance data: a mathematical modelling study. J Int AIDS Soc. 2022; 25(9):e25994. 36050916 10.1002/jia2.25994 PMC9437443

[21] Montréal signe la Déclaration de Paris visant à l'éradication du VIH/SIDA [press release]. Ville de Montréal. December 1, 2017.

[22] Stansfield SE , Heitner J , Mitchell KM , Doyle CM , Milwid RM , Moore M , et al. Population‐level impact of expanding PrEP coverage by offering long‐acting injectable PrEP to MSM in three high‐resource settings: a model comparison analysis. J Int AIDS Soc. 2023; 26(Suppl 2):e26109. 37439080 10.1002/jia2.26109 PMC10339001

[23] Kasaie P , Pennington J , Shah MS , Berry SA , German D , Flynn CP , et al. The impact of preexposure prophylaxis among men who have sex with men: an individual‐based model. J Acquir Immune Defic Syndr. 2017; 75(2):175–183. 28498144 10.1097/QAI.0000000000001354 PMC5488295

[24] MacFadden DR , Tan DH , Mishra S . Optimizing HIV pre‐exposure prophylaxis implementation among men who have sex with men in a large urban centre: a dynamic modelling study. J Int AIDS Soc. 2016; 19(1):20791. 27665722 10.7448/IAS.19.1.20791 PMC5035769

[25] Gantenberg JR , King M , Montgomery MC , Galarraga O , Prosperi M , Chan PA , et al. Improving the impact of HIV pre‐exposure prophylaxis implementation in small urban centers among men who have sex with men: an agent‐based modelling study. PLoS One. 2018; 13(7):e0199915. 29985949 10.1371/journal.pone.0199915 PMC6037355

[26] Joint United Nations Programme on HIV/AIDS (UNAIDS) . HIV prevention 2025 – road map: getting on track to end AIDS as a public health threat by 2030. Geneva; 2022.

[27] Morgan J , Ferlatte O , Salway T , Wilton J , Hull M . Awareness of, interest in, and willingness to pay for HIV pre‐exposure prophylaxis among Canadian gay, bisexual, and other men who have sex with men. Can J Public Health. 2018; 109(5–6):791–799. 29981103 10.17269/s41997-018-0090-1 PMC6964486

[28] Milwid RM , Xia Y , Doyle CM , Cox J , Lambert G , Thomas R , et al. Past dynamics of HIV transmission among men who have sex with men in Montréal, Canada: a mathematical modeling study. BMC Infect Dis. 2022; 22(1):233. 35255860 10.1186/s12879-022-07207-7 PMC8902714

[29] Eddelbuettel D . Seamless R and C++ integration with Rcpp. Springer; 2013.

[30] Eddelbuettel D , Balamuta JJ . Extending R with C++: a brief introduction to Rcpp. Am Stat. 2018; 72(1):28–36.

[31] Eddelbuettel D , Francois R . Rcpp: seamless R and C++ integration. J Stat Softw. 2011; 40(8):18.

[32] Toni T , Welch D , Strelkowa N , Ipsen A , Stumpf MPH . Approximate Bayesian computation scheme for parameter inference and model selection in dynamical systems. J R Soc Interface. 2009; 6(31):187–202. 19205079 10.1098/rsif.2008.0172 PMC2658655

[33] Mariño IP , Zaikin A , Míguez J . A comparison of Monte Carlo‐based Bayesian parameter estimation methods for stochastic models of genetic networks. PLoS One. 2017; 12(8):e0182015. 28797087 10.1371/journal.pone.0182015 PMC5552360

[34] Doyle CM , Maheu‐Giroux M , Lambert G , Mishra S , Apelian H , Messier‐Peet M , et al. Combination HIV prevention strategies among Montreal gay, bisexual, and other men who have sex with men in the PrEP era: a latent class analysis. AIDS Behav. 2021; 25(1):269–283. 32648063 10.1007/s10461-020-02965-4 PMC7846508

[35] Greenwald ZR , Maheu‐Giroux M , Szabo J , Robin JAB , Boissonnault M , Nguyen VK , et al. Cohort profile: l'Actuel Pre‐Exposure Prophylaxis (PrEP) Cohort study in Montreal, Canada. BMJ Open. 2019; 9(6):e028768. 10.1136/bmjopen-2018-028768 PMC6597624 31248931

[36] Flores Anato JL , Panagiotoglou D , Greenwald ZR , Blanchette M , Trottier C , Vaziri M , et al. Chemsex and incidence of sexually transmitted infections among Canadian pre‐exposure prophylaxis (PrEP) users in the l’Actuel PrEP Cohort (2013–2020). Sexually Transmitted Infections. 2022;98(8), 549–556. 10.1136/sextrans-2021-055215 35039437 PMC9685712

[37] Xia Y , Greenwald ZR , Milwid RM , Trottier C , Boissonnault M , Gaul N , et al. Pre‐exposure Prophylaxis Uptake Among Men Who Have Sex With Men Who Used nPEP: A Longitudinal Analysis of Attendees at a Large Sexual Health Clinic in Montréal (Canada). JAIDS Journal of Acquired Immune Deficiency Syndromes. 2020;85(4):408–415. 10.1097/qai.0000000000002472 33136737

[38] Volz E , Heckathorn DD . Probability based estimation theory for respondent driven sampling. J Off Stat. 2008; 24(1):79.

[39] Centers for Disease Control and Prevention . Preexposure prophylaxis for the prevention of HIV infection in the United States –2021 update—a clinical practice guideline. US Department of Health and Human Services; 2021.

[40] Sang JM , McAllister K , Wang L , Barath J , Lal A , Parlette A , et al. Examining provincial PrEP coverage and characterizing PrEP awareness and use among gay, bisexual and other men who have sex with men in Vancouver, Toronto and Montreal, 2017–2020. J Int AIDS Soc. 2022; 25(10):e26017. 36306245 10.1002/jia2.26017 PMC9616170

[41] Gaspar M , Tan DHS , Lachowsky N , Hull M , Wells A , Sinno J , et al. HIV pre‐exposure prophylaxis (PrEP) should be free across Canada to those meeting evidence‐based guidelines. Can J Hum Sex. 2022; 31(3):309–313.

[42] Wang L , Moqueet N , Lambert G , Grace D , Rodrigues R , Cox J , et al. Population‐level sexual mixing according to HIV status and preexposure prophylaxis use among men who have sex with men in Montreal, Canada: implications for HIV prevention. Am J Epidemiol. 2020; 189(1):44–54. 31612213 10.1093/aje/kwz231 PMC7119299

[43] Grulich AE , Bavinton BR . Scaling up preexposure prophylaxis to maximize HIV prevention impact. Curr Opin HIV AIDS. 2022; 17(4):173–178. 35762370 10.1097/COH.0000000000000739

[44] Grace D , Jollimore J , MacPherson P , Strang MJP , Tan DHS . The pre‐exposure prophylaxis‐stigma paradox: learning from Canada's first wave of PrEP users. AIDS Patient Care STDs. 2018; 32(1):24–30. 29185801 10.1089/apc.2017.0153 PMC5756933

[45] Cox J , Apelian H , Moodie EEM , Messier‐Peet M , Hart TA , Grace D , et al. Use of HIV pre‐exposure prophylaxis among urban Canadian gay, bisexual and other men who have sex with men: a cross‐sectional analysis of the Engage cohort study. CMAJ Open. 2021; 9(2):E529–E538. 10.9778/cmajo.20200198 PMC8177951 34021010

[46] National AIDS Trust . Not PrEPared: barriers to accessing HIV prevention drugs in England. 2022.

[47] Flowers P , MacDonald J , McDaid L , Nandwani R , Frankis J , Young I , et al. How can we enhance HIV pre exposure prophylaxis (PrEP) awareness and access?: recommendation development from process evaluation of a national PrEP programme using implementation science tools. medRxiv. 2022:2022.06.09.22276189.

[48] Gaspar M , Wells A , Hull M , Tan DHS , Lachowsky N , Grace D . “What other choices might I have made?”: sexual minority men, the PrEP cascade and the shifting subjective dimensions of HIV risk. Qual Health Res. 2022; 32(8–9):1315–1327. 35616240 10.1177/10497323221092701 PMC9350448

[49] Pico‐Espinosa OJ , Hull M , MacPherson P , Grace D , Lachowsky N , Gaspar M , et al. Reasons for not using pre‐exposure prophylaxis for HIV and strategies that may facilitate uptake in Ontario and British Columbia among gay, bisexual and other men who have sex with men: a cross‐sectional survey. CMAJ Open. 2023; 11(3):E560–E568. 10.9778/cmajo.20220113 PMC10310342 37369522

[50] Grace D , Gaspar M , Wells A , Sinno J , Daroya E , Montess M , et al. Injectable pre‐exposure prophylaxis for HIV prevention: perspectives on the benefits and barriers from gay, bisexual, and queer men and health system stakeholders in Ontario, Canada. AIDS Patient Care STDs. 2023;37(6):306–315. 37195728 10.1089/apc.2023.0034 PMC10280192

[51] Giguère K , Vaziri M , Olivier C , Charest L , Szabo J , Thomas R , et al. Characteristics of new HIV diagnoses over 1995–2019: A clinic‐based study in Montréal, Canada. PLOS ONE. 2021;16(10), e0258383. 10.1371/journal.pone.0258383 34618875 PMC8496787

