# Supplementary material for: Population‐level effectiveness of pre‐exposure prophylaxis for HIV prevention among men who have sex with men in Montréal (Canada): a modelling study of surveillance and survey data
Source: J Int AIDS Soc. 2023 Dec 6;26(12):e26194. doi: 10.1002/jia2.26194 (PMC10699110; doi:10.1002/jia2.26194)
Supplement: Supplementary file 1 — Figure S1. Empirical estimates of pre‐exposure prophylaxis (PrEP) eligibility among men who have sex with men (MSM) in Montréal. The estimated annual percentage of Montréal MSM eligible for PrEP (according to the provincial criteria) among Engage participants that self‐reported a negative or unknown HIV serostatus. The first four Engage study visits occurred annually over 2017‐2021. All estimates were adjusted by RDS‐II and inverse probability of censoring weights. The error bars show the estimated 95% confidence intervals. Figure S2. Empirical estimates of pre‐exposure prophylaxis (PrEP) uptake among men who have sex with men (MSM) in Montréal. The estimated percentage of Montréal MSM that reported first taking PrEP each year among Engage participants eligible for PrEP (according to the modelled criteria). No Engage participant reported first taking PrEP in 2013. To obtain estimates before 2017 (when the study began), we assumed the number eligible at baseline was constant. All estimates were adjusted by RDS‐II and inverse probability of censoring weights. The error bars show the estimated 95% confidence intervals. Figure S3. Empirical estimates of pre‐exposure prophylaxis (PrEP) adherence among men who have sex with men (MSM) in Montréal. Estimates of PrEP adherence among Montréal MSM calculated using the Engage cohort and adjusted by RDS‐II and inverse probability of censoring weights. The first four Engage study visits occurred annually over 2017‐2021. Panel A displays the self‐reported average number of pills missed per week among continuous PrEP users at each study visit. Panel B displays the self‐reported percentage of anal sex acts covered by PrEP among continuous PrEP users at the third and fourth study visits. The error bars show the estimated 95% confidence intervals. Figure S4. Empirical estimates of pre‐exposure prophylaxis (PrEP) dosing schedule among men who have sex with men (MSM) in Montréal. The estimated percentage of PrEP users following a daily [file JIA2-26-e26194-s001.pdf]

Population-level effectiveness of pre-exposure prophylaxis for HIV prevention among men who have sex with men in Montréal: a modelling study of surveillance and survey data

Supplementary Materials

Carla M Doyle[1], Rachael M Milwid[1], Joseph Cox[1,2,3], Yiqing Xia[1], Gilles Lambert[2], Cécile Tremblay[4,5], Joanne Otis[6], Marie-Claude Boily[7], Jean-Guy Baril[8,9], Sarah-Amélie Mercure[2], Réjean Thomas[10], Benoit Trottier[9], Sharmistha Mishra[11, 12, 13], Mathieu Maheu-Giroux[1§]

[1]Department of Epidemiology and Biostatistics, School of Population and Global Health, McGill University, Montréal, QC

[2]Direction Régionale de Santé Publique de Montréal, Montréal, QC

[3]Clinical Outcomes Research and Evaluation, Research Institute - McGill University Health Centre, Montréal, QC

[4]Centre de Recherche du Centre Hospitalier de l'Université de Montréal (CRCHUM), Montréal, QC

[5]Département de Microbiologie, Infectiologie et Immunologie, Université de Montréal, Montréal, QC

[6]Département de Sexologie, Université du Québec à Montréal, Montréal, QC

[7]Department of Infectious Diseases, Imperial College London, London, UK

[8]Department of Family Medicine, Centre Hospitalier de l'Université de Montréal, Montréal, QC

[9]Clinique de médecine urbaine du Quartier Latin, Montréal, QC

[10]Clinique médicale l'Actuel, Montréal, QC

[11]Department of Medicine, St. Michael's Hospital, University of Toronto, Toronto, ON

[12]Institute of Medical Sciences, University of Toronto, Toronto, ON

[13]Institute of Health Policy Management and Evaluation, Dalla Lana School of Public Health, University of Toronto, Toronto, ON

1. Pre-exposure prophylaxis (PrEP) parameterization: Additional details

Eligibility

Québec guidelines indicate PrEP for men who have sex with men (MSM) and transgender women that had condomless anal sex in the past six months and one of the following[1-3]:

1. Use of non-occupational post-exposure prophylaxis (PEP) twice or more in their lifetime.
2. Infection with syphilis or an anal bacterial sexually transmitted infection (STI) in their lifetime *(update 2019: especially if in the last 12 months).*
3. Sex with a partner living with HIV whose risk of transmission is considered high.
4. Two or more sex partners in the past six months.
5. Use of psychoactive substances during sex.

Given the scope of the model, it is not possible to match all these criteria. Criterion 2 and 5 cannot be included, as we do not model the transmission of other STIs or drug use. Criterion 3 is not included as 1) ART coverage and viral suppression levels among people living with HIV (PLHIV) in Montréal are very high, and 2) we do not model HIV status disclosure. Exclusion of this criteria in the model should have little impact as, according to l'Actuel PrEP Cohort data, fewer than 2% of MSM consulting for PrEP had sex with a partner living with HIV with a detectable viral load as their sole indication for PrEP. Thus, MSM not living with HIV were eligible for PrEP in the model if they had any anal sex acts unprotected by condoms in the past six months and either: 1) ≥2 partnerships in the past six months or 2) ≥2 lifetime uses of PEP. Altogether, 85% of MSM recommended PrEP in the l'Actuel PrEP Cohort were indicated for use based on either condomless anal sex with multiple partners or repeated PEP use. In sensitivity analyses, we assessed the impact of expanding the eligibility pool in the model (see Figure S10).

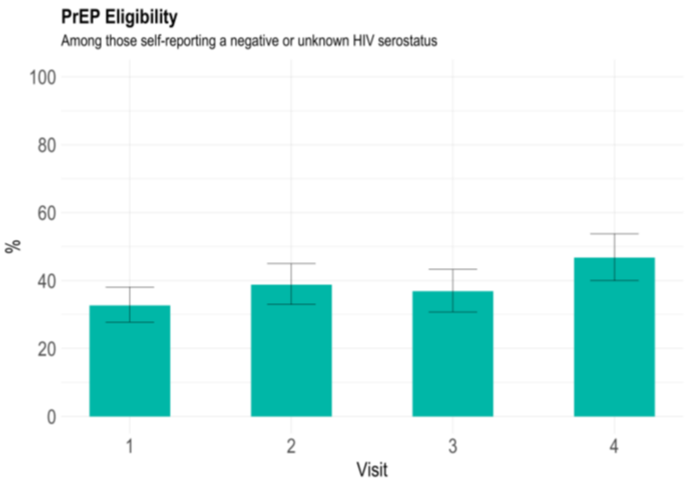

Figure S1. Empirical estimates of pre-exposure prophylaxis (PrEP) eligibility among men who have sex with men (MSM) in Montréal. The estimated annual percentage of Montréal MSM eligible for PrEP (according to the provincial criteria) among Engage participants that self-reported a negative or unknown HIV serostatus. The first four Engage study visits occurred annually over 2017-2021. All estimates were adjusted by RDS-II and inverse probability of censoring weights. The error bars show the estimated 95% confidence intervals.

Initiation

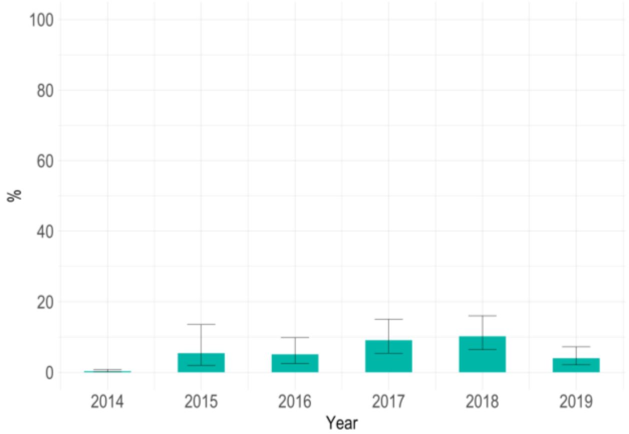

Figure S2. Empirical estimates of pre-exposure prophylaxis (PrEP) uptake among men who have sex with men (MSM) in Montréal. The estimated percentage of Montréal MSM that reported first taking PrEP each year among Engage participants eligible for PrEP (according to the modelled criteria). No Engage participant reported first taking PrEP in 2013. To obtain estimates before 2017 (when the study began), we assumed the number eligible at baseline was constant. All estimates were adjusted by RDS-II and inverse probability of censoring weights. The error bars show the estimated 95% confidence intervals.

Adherence

Engage measured adherence among continuous PrEP users (those reporting ever using PrEP continuously at baseline or in the past six months during follow-up) at all visits by capturing the self-reported number of daily doses missed per week (*"On average, how many days per week have you missed your dose of PrEP medication?"*). Additionally, a measure of PrEP-protected anal sex was included in the study questionnaire as of the third visit (*"In the past 6 months, how often were you on PrEP when you had sexual activities involving anal sex (either as top or bottom)?"*). Together, these measures indicated consistently high levels of self-reported adherence in MSM taking PrEP daily (Figure S3). However, across visits, approximately 40%-60% of participants reporting PrEP use self-reported strictly continuous use (Figure S4). Therefore, many Montréal MSM do indeed use PrEP on a situational basis. Without understanding the adherence or pill taking frequency of on-demand users in our setting, we did not model differential PrEP adherence. Instead, we parameterized PrEP effectiveness by the intention-to-treat estimate from the IPERGAY trial (which included Montréal MSM)[4]. Among all IPERGAY participants, 43% (95%CI: 35%-51%) self-reported correct use of the on-demand PrEP schedule[4]. In sensitivity analyses, we modelled an increased efficacy of 96%, corresponding to taking four doses per week, the threshold often used to define high adherence[5, 6].

A

B

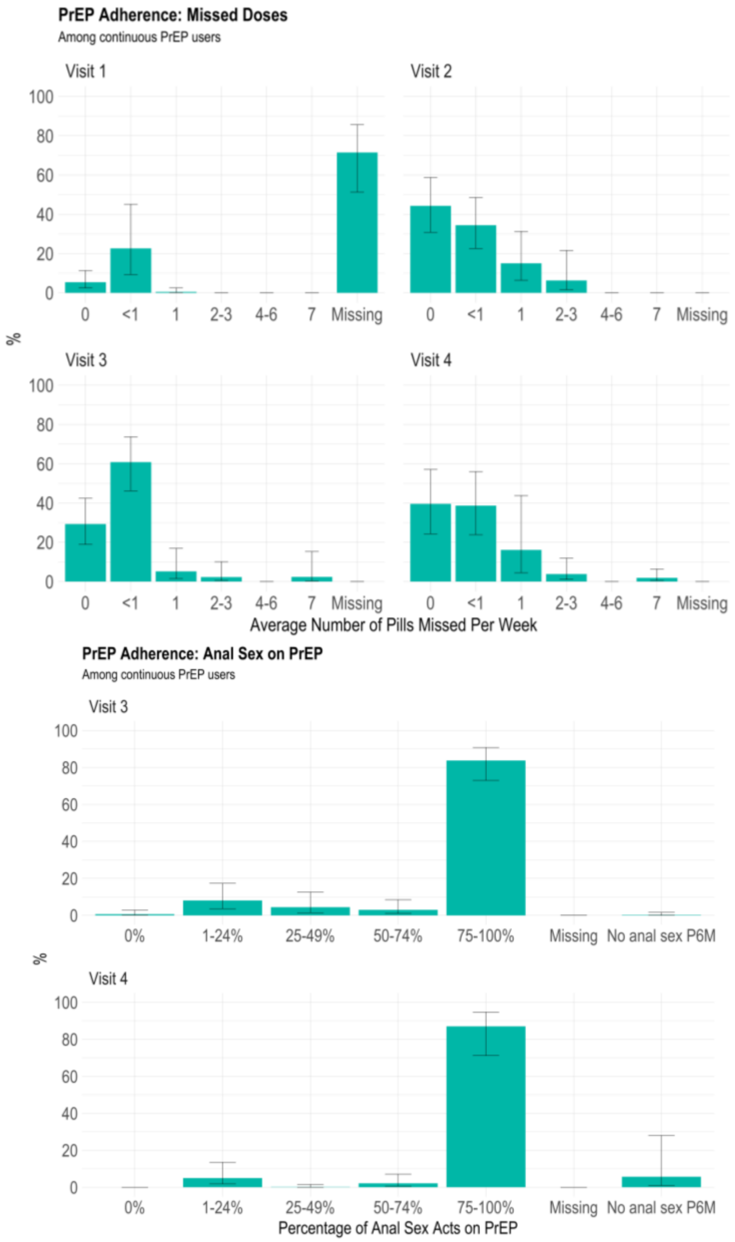

Figure S3. Empirical estimates of pre-exposure prophylaxis (PrEP) adherence among men who have sex with men (MSM) in Montréal. Estimates of PrEP adherence among Montréal MSM calculated using the Engage cohort and adjusted by RDS-II and inverse probability of censoring weights. The first four Engage study visits occurred annually over 2017-2021. Panel A displays the self-reported average number of pills missed per week among continuous PrEP users at each study visit. Panel B displays the self-reported percentage of anal sex acts covered by PrEP among continuous PrEP users at the third and fourth study visits. The error bars show the estimated 95% confidence intervals.

Persistence

The model assumes that persistence does not differ across sexual activity groups. This is supported by a recent analysis of *l'Actuel PrEP Cohort* data, which found no difference in persistence among individuals that engaged and did not engage in chemsex[7].

Schedule

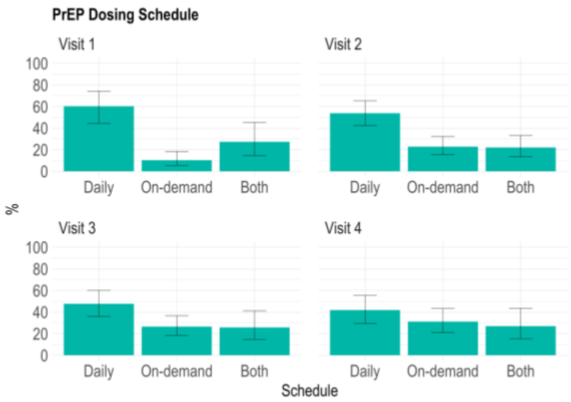

Figure S4. Empirical estimates of pre-exposure prophylaxis (PrEP) dosing schedule among men who have sex with men (MSM) in Montréal. The estimated percentage of PrEP users following a daily or on-demand dose schedule calculated using the Engage cohort and adjusted by RDS-II and inverse probability of censoring weights. The error bars show the estimated 95% confidence intervals. The first four Engage study visits occurred annually over 2017-2021.

Sexual behaviour changes

Studies of sexual behaviour changes after PrEP initiation have had mixed results[12, 13]. Among IPERGAY trial participants, which included MSM from Montréal, there was evidence that sexual risk practices remained stable up to 24 months after enrollment (data up to 2015)[14]. However, subsequent analyses of the IPERGAY trial open-label extension study showed the proportion of participants reporting not using condoms at their last anal sex act potentially increased from 77% to 86%[15]. Using *l'Actuel PrEP Cohort* data up to 2015, another article noted that while some changes might have occurred (within three months of PrEP initiation, 25%, 43%, and 32% of participants reported increases, no changes, and decreases, respectively, in the number of sexual partners and condomless anal sex following PrEP initiation), there are challenges in causally attributing behaviour changes to PrEP[16].

Despite the potential for sexual behaviour change while on PrEP, as others have noted, as PrEP is used mainly by individuals at ongoing risk of HIV, it's use outweighs the impacts of such changes[12, 17]. Indeed, no individual in the *l'Actuel PrEP Cohort* acquired HIV while taking PrEP[12, 16]. As such, we do not model any behaviour changes and do not expect this to influence our results.

2. Select Calibration Results

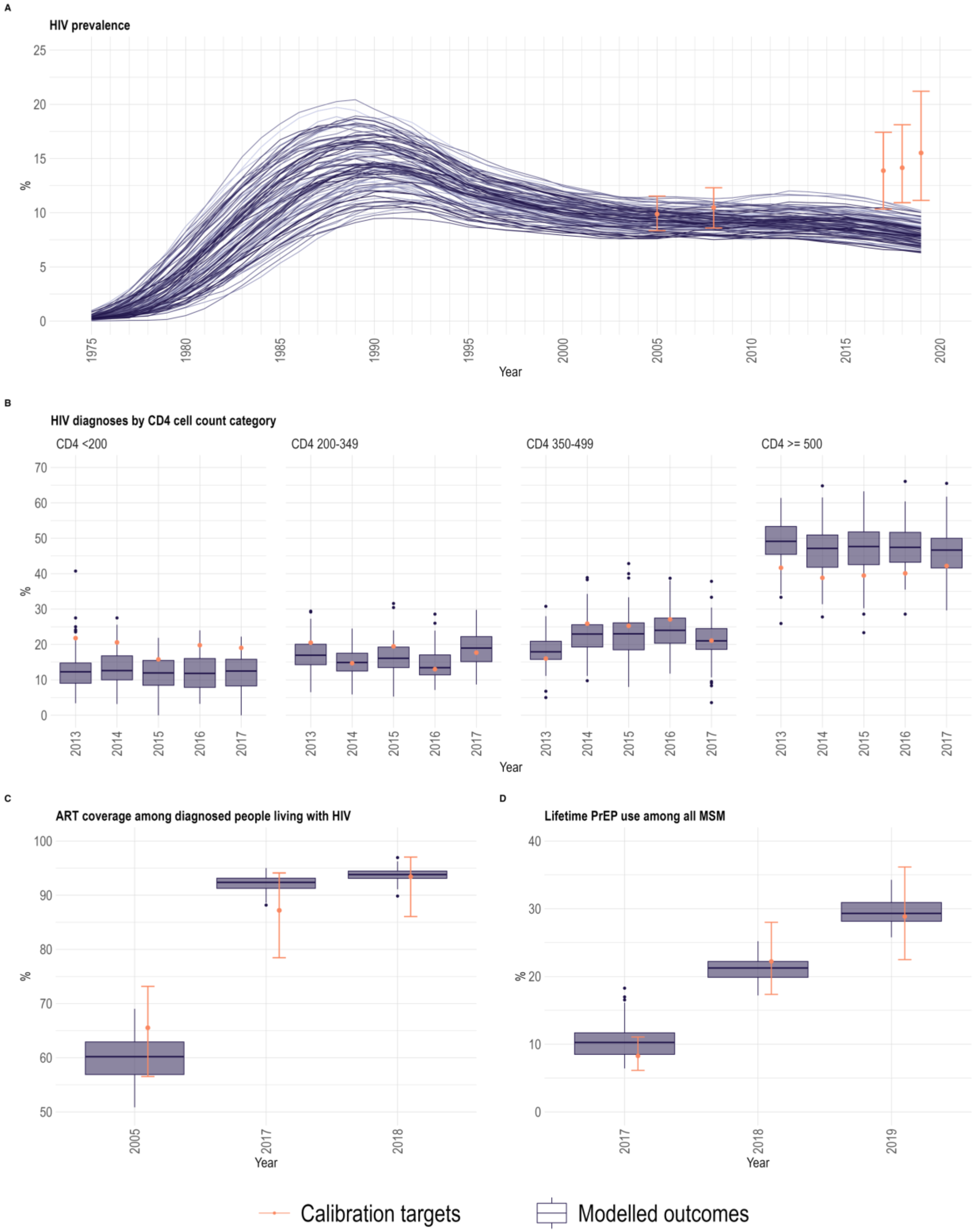

Figure S5. Select model calibration results reproduced from Milwid et al[8]. Model calibration produced 100 parameter sets, each of which were simulated once to produce figures of the HIV prevalence over 1975-2019 (panel A), the proportion of new HIV diagnoses in each CD4 cell count category over 2013-2017 (panel B), the proportion of diagnosed people living with HIV on antiretroviral treatment (ART) in 2005,

2017, and 2018 (panel C), and the proportion that ever used pre-exposure prophylaxis (PrEP) over 2017-2019. The purple lines boxplots show the model simulations. The orange points and error bars show the target data used in calibration. For results of the remaining calibration outcomes, please refer to the supplementary materials of our previous model publication by Milwid et al[8].

3. Additional Model Results

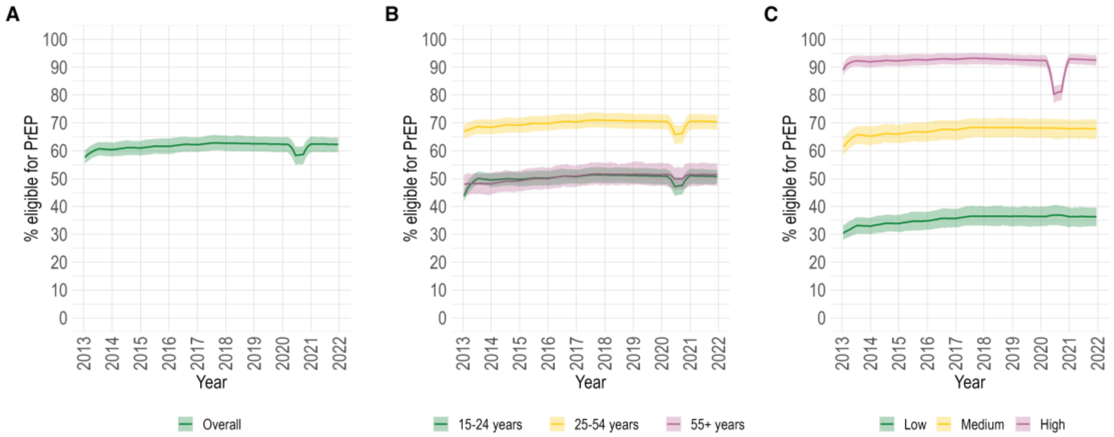

Figure S6. Modelled pre-exposure prophylaxis (PrEP) eligibility among men who have sex with men (MSM) not living with HIV in Montréal. The model estimated percentage of MSM not living with HIV eligible for PrEP over 2013-2021 in Montréal: overall (panel A) and stratified by age (panel B) and sexual activity group (panel C). The coloured lines and bands show the model posterior mean and 90% credible intervals, respectively.

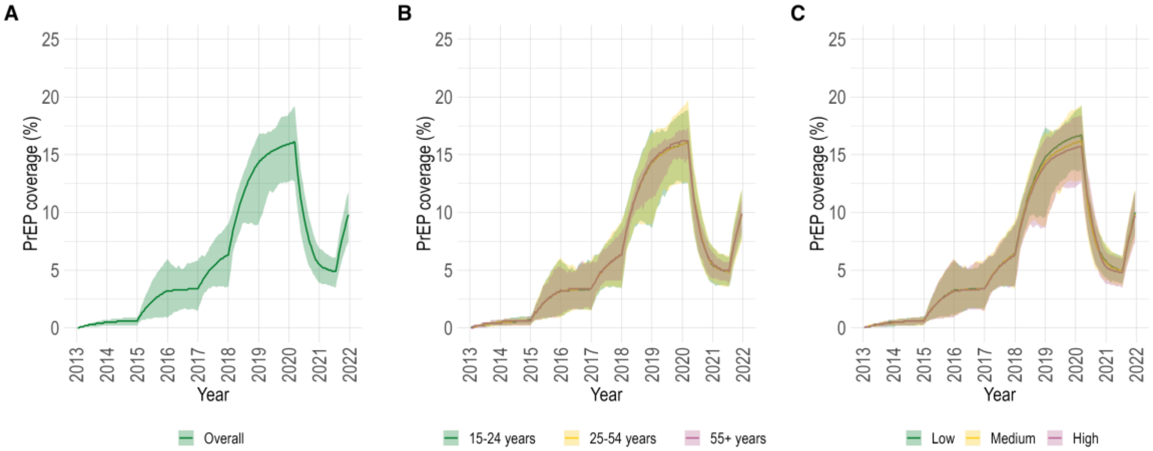

Figure S7. Modelled pre-exposure prophylaxis (PrEP) coverage among PrEP-eligible men who have sex with men (MSM) not living with HIV in Montréal. The model estimated PrEP coverage over 2013-2021 among MSM eligible for PrEP in Montréal: overall (panel A) and stratified by age (panel B) and sexual activity group (panel C). The coloured lines and bands show the model posterior mean and 90% credible intervals, respectively.

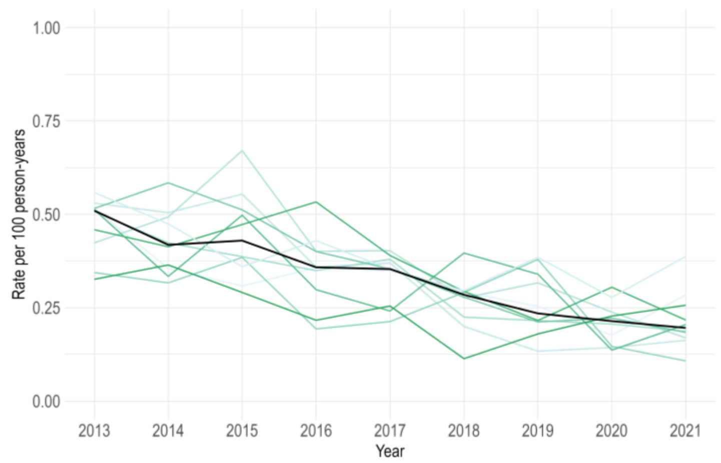

Figure S8. Annual HIV incidence across ten simulations of one parameter set. Estimated HIV incidence rates over 2013-2021 among men who have sex with men (MSM) in Montréal under the provincial pre-exposure prophylaxis (PrEP) intervention scenario. The green lines display the results per simulation and the black line displays the median.

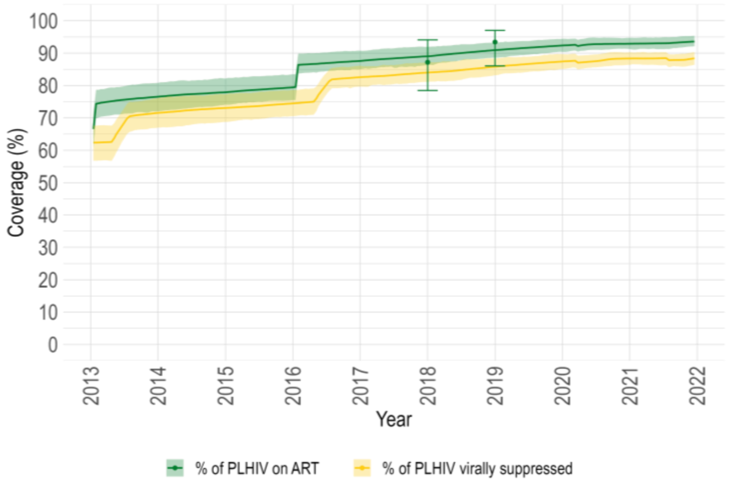

Figure S9. Modelled antiretroviral treatment (ART) and viral load suppression coverage among men who have sex with men (MSM) living with HIV (PLHIV) in Montréal. The model ART and viral load suppression coverage among MSM living with HIV over 2013-2021. From 2013 onward, all PLHIV in the model are eligible for ART and initiate upon HIV diagnosis. The coloured lines and bands show the model posterior mean and 90% credible intervals, respectively. The two points and bars display the estimated ART coverage and 95% confidence intervals calculated from *Engage* and adjusted by RDS-II and inverse probability of censoring weights.

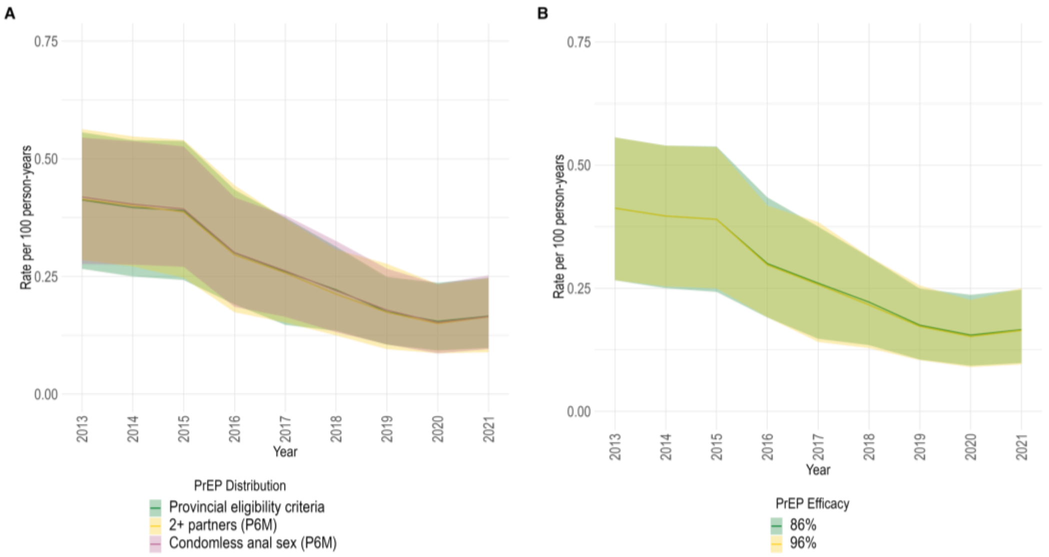

Figure S10. Sensitivity Analyses. The model estimated HIV incidence rates over 2013-2021 among Montréal men who have sex with men (MSM) under the provincial pre-exposure prophylaxis (PrEP) intervention scenario with different PrEP-eligibility criteria (panel A) and with different PrEP efficacies (Panel B). The coloured lines and bands show the posterior median and 90% credible intervals, respectively.

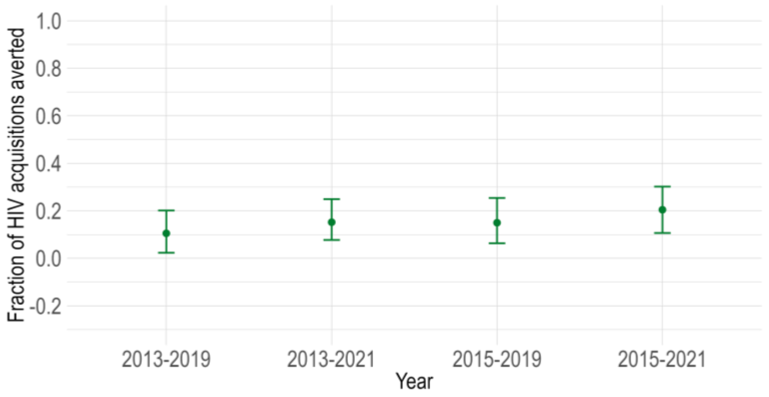

Figure S11. Cumulative Acquisitions Averted. Estimated cumulative fraction of acquisitions averted due to pre-exposure prophylaxis (PrEP) intervention among men who have sex with men (MSM) in Montréal (provincial PrEP intervention scenario) over varying time periods. The coloured points and bars show the posterior mean and 90% credible intervals, respectively.

Table S1. Annual pre-exposure prophylaxis (PrEP) impact evaluation results among men who have sex with men in Montréal over 2015-2021 in the model population and scaled to the total population.

Year	Estimated size of MSM population in Montréal* N	Number of HIV acquisitions averted in the total MSM population N (90%CrI)
Provincial PrEP intervention scenario		
2015	49,553	7 (-28-40)
2016	49,761	10 (-17-36)
2017	50,440	19 (-4-50)

2018	51,401	30 (6-60)
2019	52,558	49 (20-88)
2020	52,895	44 (14-81)
2021	51,947	50 (11-94)

Abbreviations: Men who have sex with men (MSM), pre-exposure prophylaxis (PrEP), credible interval (CrI).

*Taken as 6.1% of the total male population in Montréal[9], as given in the Institut de la statistique du Québec (ISQ) population size estimates (2015–2021) of the Montréal administrative region[10].

4. Supplement References

1. Ministère de la Santé et des Services sociaux. Avis intérimaire sur la prophylaxie préexposition au virus de l'immunodéficience humaine. Gouvernment du Quebec; 2013.
2. Ministère de la Santé et des Services sociaux. La prophylaxie préexposition au virus de l'immunodéficience humaine: Guide pour les professionnels de la santé du Québec. Gouvernement du Québec,; 2017 November.
3. Ministère de la Santé et des Services sociaux. La prophylaxie préexposition au virus de l'immunodéficience humaine: Guide pour les professionnels de la santé du Québec. Gouvernement du Québec,; 2019 January.
4. Molina JM, Capitant C, Spire B, Pialoux G, Cotte L, Charreau I, et al. On-Demand Preexposure Prophylaxis in Men at High Risk for HIV-1 Infection. The New England journal of medicine. 2015;373(23):2237-46.
5. Anderson PL, Glidden DV, Liu A, Buchbinder S, Lama JR, Guanira JV, et al. Emtricitabine-tenofovir concentrations and pre-exposure prophylaxis efficacy in men who have sex with men. Sci Transl Med. 2012;4(151):151ra25.
6. Zhang J, Li C, Xu J, Hu Z, Rutstein SE, Tucker JD, et al. Discontinuation, suboptimal adherence, and reinitiation of oral HIV pre-exposure prophylaxis: a global systematic review and meta-analysis. The lancet HIV. 2022;9(4):e254-e68.
7. Flores Anato JL, Panagiotoglou D, Greenwald ZR, Trottier C, Vaziri M, Thomas R, et al. Chemsex practices and pre-exposure prophylaxis (PrEP) trajectories among individuals consulting for PrEP at a large sexual health clinic in Montréal, Canada (2013-2020). Drug Alcohol Depend. 2021;226:108875.
8. Milwid RM, Xia Y, Doyle CM, Cox J, Lambert G, Thomas R, et al. Past dynamics of HIV transmission among men who have sex with men in Montréal, Canada: a mathematical modeling study. BMC Infect Dis. 2022;22(1):233.
9. Institut de la statistique du Québec. Enquête québécoise sur la santé de la population, 2020-2021. 2023.
10. Estimations de la population des régions administratives selon le groupe d'âge et le sexe, âge médian et âge moyen, Québec, 1er juillet 1996 à 2021 [Internet]. 2022 [cited May 29, 2022]. Available from: https://statistique.quebec.ca/en/document/population-and-age-and-sex-structure-administrative-regions/tableau/population-age-sex-median-administrative-regions-quebec#tri_tertr=06&tri_pop=10.
